# MiR-199-3p enhances muscle regeneration and ameliorates aged muscle and muscular dystrophy

**DOI:** 10.1038/s42003-021-01952-2

**Published:** 2021-03-29

**Authors:** Masashi Fukuoka, Hiromi Fujita, Kosumo Numao, Yasuko Nakamura, Hideo Shimizu, Masayuki Sekiguchi, Hirohiko Hohjoh

**Affiliations:** 1 Department of Molecular Pharmacology, National Institute of Neuroscience, NCNP, Tokyo, Japan; 2 Department of Degenerative Neurological Diseases, National Institute of Neuroscience, NCNP, Tokyo, Japan; 3 Present Address: Department of Biological Science and Technology, Faculty of Industrial Science and Technology, Tokyo University of Science, Tokyo, Japan

**Keywords:** Biomarkers, Biomarkers, miRNAs

## Abstract

Parabiosis experiments suggest that molecular factors related to rejuvenation and aging circulate in the blood. Here, we show that miR-199-3p, which circulates in the blood as a cell-free miRNA, is significantly decreased in the blood of aged mice compared to young mice; and miR-199-3p has the ability to enhance myogenic differentiation and muscle regeneration. Administration of miR-199 mimics, which supply miR-199-3p, to aged mice resulted in muscle fiber hypertrophy and delayed loss of muscle strength. Systemic administration of miR-199 mimics to mdx mice, a well-known animal model of Duchenne muscular dystrophy (DMD), markedly improved the muscle strength of mice. Taken together, cell-free miR-199-3p in the blood may have an anti-aging effect such as a hypertrophic effect in aged muscle fibers and could have potential as a novel RNA therapeutic for DMD as well as age-related diseases. The findings provide us with new insights into blood-circulating miRNAs as age-related molecules.

## Abstract (web summary)

Fukuoka et al. investigate the possible role of miR-199-3p during aging, which significantly decreases in the blood of aged mice compared to young mice. They also show that miR-199-3p enhance muscle regeneration, and administering miR-199 mimics to mdx mice, a dystrophy animal model, markedly improves muscle strength of the mice, highlighting its therapeutic potential.

## Introduction

Vital functions, such as muscle force, operation of the nervous system, respiratory function, defense against infection, and resistance against diseases, decline with aging. Diseases and dysfunctions associated with such functional decline are increasing and becoming major problems in aging societies. Various changes, such as metabolic and gene regulation changes, occur in the body with aging^1–3^; and studying such changes may help to understand aging and provide strategies to deal with the problems facing aging societies. Biomolecules (including genes) related to aging have been identified and may be useful in aging research; e.g., β-galactosidase and p16^lnk4a^ expression are associated with cellular senescence^4–6^, and a mouse model lacking the *klotho* gene is known as an aging model with a number of phenotypes, resembling human premature aging^7^.

Parabiosis is the union between two individuals that share a common vascular system, and can be generated experimentally by surgery^8^. Experiments with parabiosis, in which young and aged rodents were conjoined to share a common vascular system, showed that the aging process in young animals was accelerated, while aged animals were rejuvenated^9–12^. The findings strongly suggest that certain factors related to rejuvenation and aging are present, and circulating with blood in the vascular system; however, such circulating factors are not yet fully understood^13^.

MicroRNAs (miRNAs) are 21–23-nucleotide-long small noncoding RNAs, which are processed from longer transcripts (primary miRNAs) by digestion, with a microprocessor complex in the nucleus and Dicer in the cytoplasm, and incorporated into the RNA-induced silencing complex (RISC)^14,15^. MiRNA in RISC functions as a mediator in gene silencing, where translation of messenger RNAs (mRNAs) having partial complementarity to the miRNA is inhibited or mRNAs carrying nearly complementary sequences to the miRNA are digested^14,15^. Thus, miRNAs play important roles in the gene regulation via suppressing their target gene expression. Thousands of miRNA genes have been found in animals and plants [see the microRNA database (miRBase): http://www.mirbase.org/index.shtml]. The expression profile of miRNAs has been examined, and tissue- and developmental stage-specific expression and disease-associated expression of miRNAs have been detected^16–21^. Gene regulation involving miRNAs is closely related to various vital functions and life phenomena, including diseases^14,16,21–24^.

MiRNAs are present outside, as well as inside the cells^25^. For example, miRNAs are present in the blood as cell-free nucleotides and circulate in the vascular system^26,27^. Such cell-free miRNAs (cf-miRNAs) are incorporated into small vesicles called extracellular vesicles or associated with proteins, thereby gaining protection from extracellular nucleases^25,28^. The presence of some cf-miRNAs may be significant; for example, significant associations between cf-miRNAs and certain diseases have been detected^26,27,29–31^. Cf-miRNAs may reflect changes in the physical condition; thus, such miRNAs may be potential biomarkers for monitoring life maintenance systems, as well as diseases.

The present study was conducted to examine associations between cf-miRNAs and aging, and cf-miRNAs in the blood of young and aged mice were investigated. The results revealed changes in cf-miRNAs between young and aged mouse blood. Among such miRNAs, miR-199-3p was markedly decreased in aged blood compared to young blood. Interestingly, miR-199-3p has the ability to enhance muscle differentiation and regeneration, and its administration to aged mice resulted in the muscle fiber expansion. When miR-199-3p was introduced into mdx mice, an animal model of Duchenne muscular dystrophy (DMD)^32^, the muscle strength of mice was markedly improved. The present study provides new insights into blood-circulating cf-miRNAs in terms of aging, latent ability, and medical applications.

## Results

### Cell-free miRNAs in the blood of young and aged mice

**Fig. 1:**
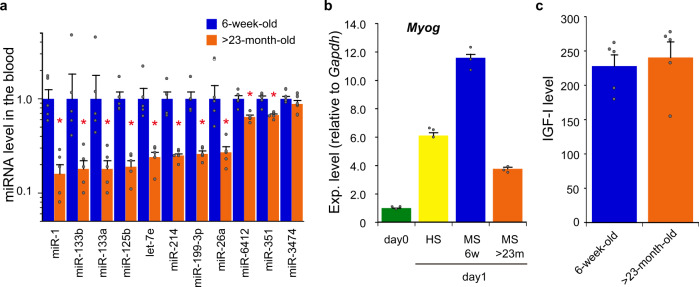
Blood-circulating substances in young and aged mice. **a** Cell-free miRNAs (cf-miRNAs) circulating with blood in young and aged mice. RNA was isolated from the plasma of young (6-week-old) and aged (>23-month-old) C57BL/6J mice, and miRNAs (indicated) were examined by qRT-PCR. The data were analyzed by the delta–delta Ct method using the data of cel-miR-39 as an external control and normalized with the data obtained from young mice as 1. Data are shown as mean ± SEM (*n* = 5 mice; *p < 0.05 by Wilcoxon rank-sum test). **b** Myogenic differentiation with young and aged mouse serum. C2C12 cells were cultured for 24 h in following the differentiation media: DMEM supplemented with 5% young mouse serum (MS, 6w) or 5% aged mouse serum (MS, >23m), and DMEM supplemented with 2% house serum (HS) as a conventional differentiation medium. Total RNA was extracted from the cells and undifferentiated C2C12 cells (day 0) as a control. The expression of *Myog* as a myogenic differentiation marker was examined by qRT-PCR and analyzed by the delta–delta Ct method, using the data of *Gapdh* as an internal reference. The data were further normalized with the data of undifferentiated cells (day 0) as 1. Data are shown as mean ± SEM (*n* = 3 independent determinations). **c** IGF-I level in young and aged mouse blood. Plasma samples were prepared from the blood of young and aged mice, and plasma levels of IGF-I were measured by ELISA. Data are shown as mean ± SEM (*n* = 5 mice).

We investigated cf-miRNAs in the blood of young (6-week-old) and aged (>23-month-old) C57BL/6J mice, using DNA microarray to identify age-related differences. The profile of cf-miRNAs exhibited marked differences between young and aged mice, i.e., cf-miRNAs that are biased toward young and aged mouse blood were detected (Table 1). Of such miRNAs, myogenic miRNAs (e.g., miR-1 and miR-133)^18,33^ were present in the miRNA group that is abundant in young blood; in other words, myogenic miRNAs were markedly less in aged blood (Table 1). The difference in the miRNAs between young and aged blood was confirmed by quantitative reverse transcription-polymerase chain reaction (qRT-PCR; Fig. 1a), and further reproduced using RNA sources extracted from small vesicles, so called exosomes, isolated from the plasma (Supplementary Fig. 1).

**Table 1:** Difference in the level of cf-miRNAs between young and aged mouse blood.

miRNA	Young Ex1, Ex2 (Ave)	Aged Ex1, Ex2 (Ave)	FC (aged/young)
mmu-miR-1a-3p	525.0, 214.8 (369.9)	56.8, 159.2 (108.0)	0.29
mmu-miR-133b-3p	506.9, 186.8 (346.9)	91.0, 177.2 (134.1)	0.39
mmu-miR-214-3p	27.1, 21.8 (24.4)	9.0, 10.6 (9.8)	0.40
mmu-miR-133a-3p	362.6, 155.9 (259.2)	67.8, 142.2 (105.0)	0.40
mmu-miR-3474	425.9, 240.9 (333.4)	79.3, 198.6 (139.0)	0.42
mmu-miR-351-5p	26.7, 27.2 (27.0)	10.8, 12.9 (11.9)	0.44
mmu-miR-6412	62.9, 60.6 (61.7)	26.2, 28.5 (27.4)	0.44
mmu-let-7e-5p	65.3, 27.2 (46.2)	17.1, 26.0 (21.6)	0.47
mmu-miR-199-3p	123.6, 72.8 (98.2)	46.0, 46.2 (46.1)	0.47
mmu-miR-125b-5p	478.3, 281.2 (379.8)	170.2, 187.1 (178.6)	0.47
mmu-miR-6364	15.1, 25.3 (20.2)	7.8, 11.5 (9.6)	0.48
mmu-miR-26a-5p	269.9, 222.7 (246.3)	82.6, 163.1 (122.8)	0.50
mmu-miR-721	10.8, 8.9 (9.8)	20.8, 20.2 (20.5)	2.08
mmu-miR-346-3p	296.7, 134.9 (215.8)	560.4, 340.0 (450.2)	2.09
mmu-miR-486-3p	484.5, 121.3 (302.9)	836.1, 437.1 (636.6)	2.10
mmu-miR-1224-3p	31.6, 10.7 (21.1)	35.3, 54.2 (44.8)	2.12
mmu-miR-5621-5p	272.2, 94.5 (183.4)	496.8, 286.7 (391.8)	2.14
mmu-miR-5620-5p	282.7, 154.4 (218.5)	573.3, 364.4 (468.9)	2.15
mmu-miR-5129-5p	47.3, 10.2 (28.8)	70.2, 53.8 (62.0)	2.16
mmu-miR-223-3p	269.1, 141.4 (205.2)	429.9, 499.2 (464.5)	2.26
mmu-miR-1900	72.8, 29.1 (50.9)	120.3, 117.5 (118.9)	2.33
mmu-miR-770-3p	102.6, 39.3 (70.9)	272.4, 131.5 (202.0)	2.85
mmu-miR-744-5p	716.4, 242.7 (479.5)	2129.1, 659.8 (1394.4)	2.91
mmu-miR-680	553.6, 277.1 (415.3)	1567.2, 888.0 (1227.6)	2.96

Signal intensities obtained from duplicated microarray analyses (experiments 1 and 2: Ex1, Ex2) and averages in parentheses (Ave) are indicated.

Fold changes (FC) in the averaged level of cf-miRNAs between aged and young mouse blood are calculated.

The top 12 cf-miRNAs from the lowest FC and highest FC are indicated.

Cell culture experiments, on the other hand, revealed that young mouse serum had the ability to induce myogenic differentiation, and the ability was stronger than that of aged mice (Fig. 1b). Insulin-like growth factor I (IGF-I), which is a major factor capable of inducing myogenic differentiation in the blood^34,35^, was examined; however, no significant difference was observed between young and aged blood (Fig. 1c). Thus, factors other than IGF-I in young blood may be implicated in myogenic differentiation.

### miR-199-3p is capable of strongly inducing myogenic differentiation

**Fig. 2:**
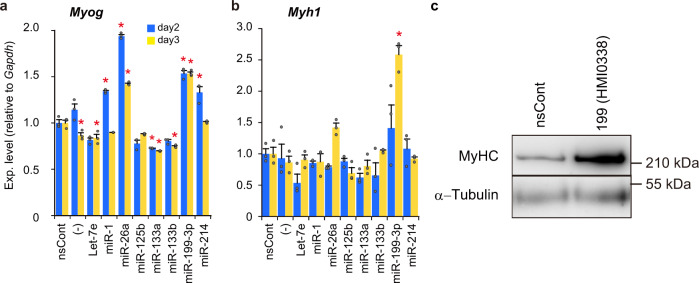
Effects of miRNAs on myogenic differentiation. **a**, **b** Expression of myogenic differentiation marker genes. MiRNAs (indicated) that are more abundant in young mouse blood than in aged mouse blood were examined. Synthetic miRNAs (Mission® microRNA mimics) were introduced into C2C12 cells; and 2 and 3 days after miRNA introduction, the expression of *Myog* (**a**) and *Myh1* (**b**) was examined by qRT-PCR as in Fig. 1b. Cells treated with non-silencing control RNAs (nsCont) and untreated cells (−) were also examined as a control. Normalized data were further normalized with the data obtained from cells treated with nsCont as 1. Data are shown as mean ± SEM (*n* = 3 independent determinations; *p < 0.001 by Dunnett’s test). **c** Myosin heavy chain (MyHC) expression. C2C12 cells transfected with Mission miR-199-3p mimic [199(HMI0338)] and nsCont were cultured for 4 days. Cell lysate was prepared and examined by western blotting using antibodies against MyHC and α-tubulin (as a loading control).

We investigated the effects of miRNAs abundant in young blood on myogenic differentiation. Synthetic miRNA mimics were introduced into C2C12 cells, a mouse myoblast cell line, and the expression of myogenic differentiation markers, the *myogenin* (*Myog*) and *myosin heavy chain 1* (*Myh1*) genes, was examined. The results were unexpected and attracted our interest: miR-199-3p, which was predicted as a negative miRNA due to the little information on myogenic differentiation, markedly induced the expression of *Myog* and *Myh1* (Fig. 2a, b). In addition, the induction of miR-199-3p was stronger than that of conventional myogenic miRNAs: the expression of myosin heavy chain in the presence of miR-199-3p was particularly remarkable (Fig. 2b, c).

To clarify the relationship between miR-199-3p and myogenic differentiation, we investigated cellular and extracellular (exosomal) miR-199-3p during myogenic differentiation, and also the effects of miR-199-3p inhibitors on the differentiation. The expression of endogenous (cellular) miR-199-3p was upregulated after the initiation of differentiation. Exosomal miR-199-3p was increased once and decreased thereafter (Supplementary Fig. 2a). Inhibitor treatment suppressed the expression of myogenic differentiation markers, *Myog*, *Myh1*, and *creatine kinase* (*Ckm*) (Supplementary Fig. 2b). Thus, the findings suggest that miR-199-3p participates in myogenic differentiation.

### Designing miR-199 mimics

We designed miR-199 mimics such that the mimics can efficiently produce miR-199-3p as a functional mediator (guide strand) in gene silencing. The designed miRNA mimics were screened and miR199#4 was selected as a competent miR-199 mimic (Supplementary Fig. 3). Subsequently, miR199#4 was used in both in vivo and in vitro experiments.

### Target genes of miR-199-3p in myogenic differentiation

**Fig. 3:**
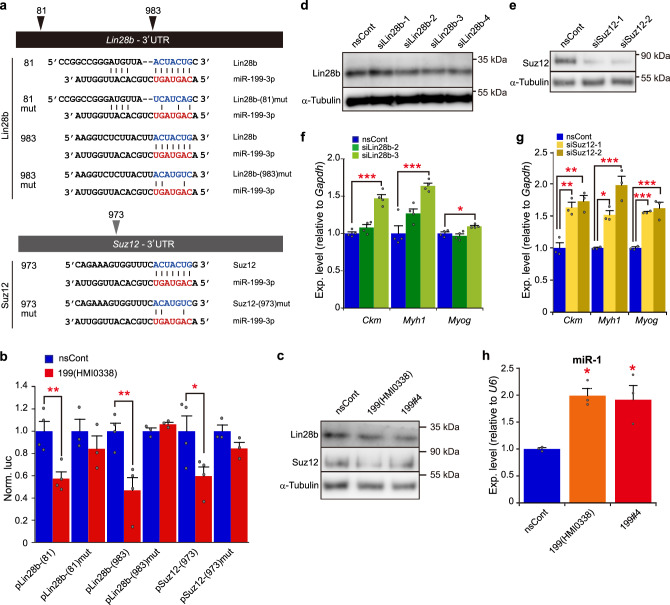
Target genes for miR-199-3p. **a** Schematic representation of predicted binding sequences of target genes. *Lin28b* and *Suz12* are candidate target genes for miR-199-3p. The predicted binding sequences and their position are shown. The seed sequence of miR-199-3p is indicated in red. Designed binding sequences with mismatches are also shown. **b** Suppression of reporter genes carrying the predicted binding sequences by miR-199-3p. Reporter plasmids, pLin28b-(81), pLin28b-(983), and pSuz12-(973), encoding the predicted binding sequences (indicated in **a**) in the 3′UTR of the *Renilla luciferase* reporter gene, and their mutant plasmids, pLin28b-(81)mut, pLin28b-(983)mut, and pSuz12-(973)mut, carrying the mismatched sequences (indicated in **a**) were co-transfected with Mission miR-199-3p mimic [199(HMI0338)] or nsCont to C2C12 cells. The *Renilla* *luciferase* activity (test reporter) was normalized with the *Photinus luciferase* (control reporter) activity. The data were further normalized to the data obtained with nsCont as 1. Data are shown as mean ± SEM [*n* = at least 3 independent determinations; *p < 0.05, **p < 0.01 by Student’s *t* test (two-tailed)]. **c** Suppression of Lin28b and Suz12 by miR-199-3p. C2C12 cells were transfected with miR-199 mimics [199(HMI0338) and our designed miR199#4 (199#4); see Supplementary Fig. 3] and nsCont. Two days later, Lin28b and Suz12 were examined by western blotting. Alpha-tubulin was examined as a loading control. **d**, **e** Gene silencing of *Lin28b* and *Suz12* by RNAi. C2C12 cells were transfected with designed siRNAs against *Lin28b* (four siRNAs) and *Suz12* (two siRNAs). Two days later, Lin28b (**d**) and Suz12 (**e**) were examined by western blotting as in **c**. **f**, **g** Expression of myogenic differentiation markers under *Lin28b* and *Suz12* gene silencing. The expression of myogenic differentiation maker genes (indicated) under *Lin28b* (**f**) and *Suz12* (**g**) gene silencing was examined by qRT-PCR and analyzed as in Fig. 2a, b. Data are shown as mean ± SEM (n = at least 3 independent determinations; *p < 0.05, **p < 0.01, ***p < 0.001 by Dunnett’s test). **h** Production of matured miR-1. C2C12 cells were transfected with miR-199 mimics and nsCont. The level of matured miR-1 in the treated cells was examined by qRT-PCR followed by the delta–delta Ct analysis, using the data of *U6 snRNA* as an internal reference. The data were normalized with the data of nsCont as 1. Data are shown as mean ± SEM (*n* = 3 independent determinations; *p < 0.05 by Dunnett’s test).

To identify target genes for miR-199-3p under myogenic differentiation, we searched for candidate genes from the TargetScan database and focused on the *Lin28b* and *Suz12* genes, both of which are expressed in C2C12 myoblasts. *Lin28b* and *Suz12* possess putative miR-199-3p binding sequences in their 3′ untranslated region (UTR; Fig. 3a). We demonstrated that the putative sequences are authentic binding sites for miR-199-3p, using the *Luciferase* reporter genes carrying the binding sequences and corresponding mismatched sequences (Fig. 3a, b). The *Luciferase* genes carrying the putative binding sequences were significantly suppressed by miR-199-3p [pLin28b-(81), pLin28-(983), and pSuz12-(973)], whereas the mismatched sequences, which possess base substitution mutations in the seed region, abrogated its suppression effect [pLin28b-(81)mut, pLin28-(983)mut, and pSuz12-(973)mut]. When miR-199 mimics were introduced to C2C12 cells, the expression of Lin28b and Suz12 was consistently suppressed (Fig. 3c). To assess whether the suppression of Lin28b and/or Suz12 directly contributes to myogenic differentiation, specific inhibition of *Lin28b* and *Suz12* was carried out by gene silencing, using RNA interference (RNAi; Fig. 3d, e). As expected, the expression of myogenic differentiation markers, such as *Ckm*, *Myh1*, and *Myog* was upregulated under *Lin28b* and *Suz12* gene silencing (Fig. 3f, g).

Since Lin28b is an RNA-binding protein involved in processing of miRNAs^36,37^, we investigated the effects of miR-199 mimics via Lin28b on myogenic miRNA processing. The level of miR-1, a major myogenic miRNA, was examined because processing of its precursor is regulated by Lin28b^37^. Matured miR-1 was significantly increased by miR-199 mimic treatment (Fig. 3h), suggesting that miR-199-3p can regulate the functional expression of miR-1 through suppression of its target *Lin28b*. Taken together, the findings suggest that miR-199-3p is involved in myogenic differentiation through suppression of its target genes, *Lin28b* and *Suz12*.

### Enhancement of muscle regeneration by miR199#4 in muscle injury mice

**Fig. 4:**
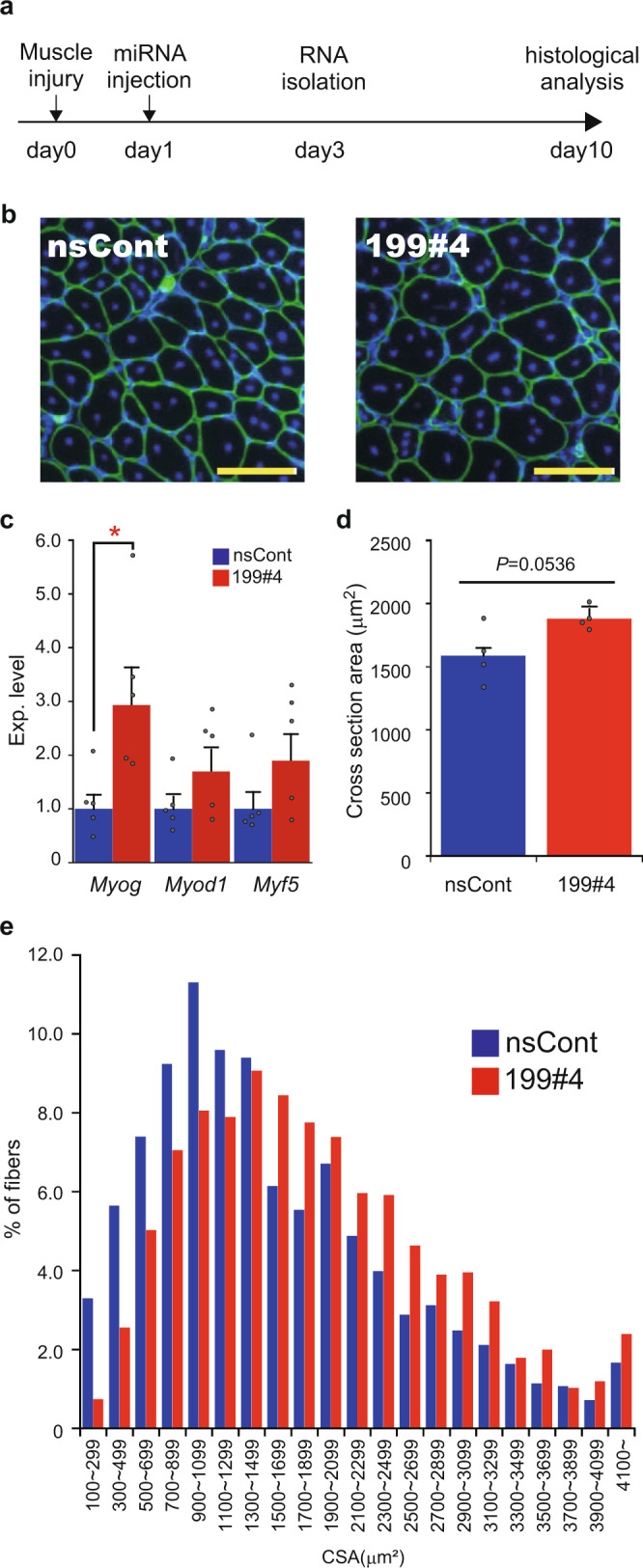
Effects of miR199#4 on muscle regeneration. **a** Schematic representation of experiment design. C57BL/6J mice (8-week-old) are injected with BaCl_2_ to the TA muscle for muscle injury (day 0), and miR199#4 and nsCont were administered to the damaged sites 24 h later (day 1). Gene expression analysis and histological analysis are performed on day 3 and day 10, respectively. **b** Photos of the cross-section of myofibers treated with miR199#4 (199#4) and nsCont. Cryosections were prepared from regenerating TA muscles and stained with an antibody against Laminin-α2 (green) and DAPI (blue). Scale bars indicate 100 µm. **c** Expression of myogenic differentiation markers. Total RNA was prepared from regenerating TA muscles on day 3, and examined by qRT-PCR for myogenic differentiation maker genes (indicated). The data were analyzed by the delta–delta Ct method, using the data of *Actb* as an internal reference and further normalized with the data obtained with nsCont as 1. Data are shown as mean ± SEM [*n* = 5 treated mice; *p < 0.05 by Student’s *t* test (two-tailed)]. **d** Averaged cross-sectional area of regenerating myofibers. Cryosections of regenerating myofibers were prepared from four mice in each miR199#4 (199#4) and nsCont treatment and examined as in **b**. The cross-sectional areas (CSAs) of regenerating myofibers, which possess central nuclei (see **b**), were measured by the ImageJ software. At least 200 CSAs per test mouse were examined and averaged, and the averaged CSAs were further averaged in each treatment group (miR199#4 and nsCont). Data are shown as mean ± SEM [*n* = 4 treated mice, Student’s *t* test (two-tailed)]. **e** Histogram of the CSAs of myofibers. The CSAs examined in **d** were fractionated by size (indicated), and the number of the CSA data in each fraction was calculated by percentage (%).

We examined the effects of an miR-199 mimic (miR199#4) in vivo using muscle injury models (Fig. 4a, b). Eight-week-old C57BL/6J mice were injected with BaCl_2_ to the tibialis anterior (TA) to induce muscle injury, and miR199#4 was administered to the damaged sites 24 h later. Two days after miR199#4 administration, the expression of myogenic marker genes was examined. *Myog* expression was significantly increased and *myogenic differentiation 1* (*Myod1*) and *myogenic factor 5* (*Myf5*) were also upregulated by miR199#4 administration (Fig. 4c). The data are consistent with the in vitro results described above (Fig. 2).

Immunohistochemical analysis of regenerating muscles was performed 9 days after miR199#4 administration. The cross-sectional areas of regenerating muscle fibers (myofibers), characterized by central nuclei, were examined (Fig. 4b). The mean cross-sectional area of regenerating myofibers treated with miR199#4 was larger than that of control myofibers treated with a non-silencing control RNA duplex (nsCont; Fig. 4d), with the difference approaching statistical significance. In addition to the mean cross-sectional area, the size-divided histogram of the cross-sectional area showed an increase in enlarged myofibers in the presence of miR199#4 (Fig. 4e).

### Hypertrophic effect of miR199#4 on aged muscle fibers

**Fig. 5:**
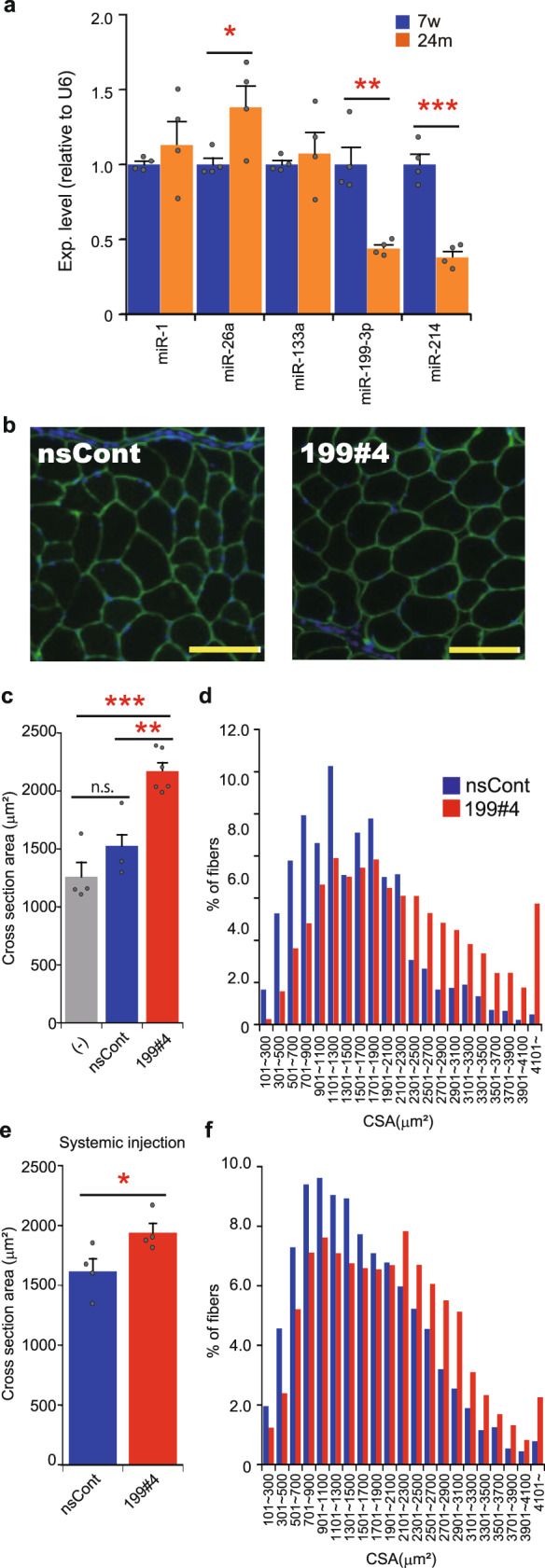
Effects of miR199#4 on aged mice. **a** Expression of miRNAs in young and aged TA muscles. The expression levels of miRNAs (indicated) in the TA muscle of young (7-week-old: 7w) and aged (~24-month-old: 24m) C57BL/6J mice were examined by qRT-PCR and analyzed by the delta–delta Ct method, using the data of *U6 snRNA* as an internal reference. The data were normalized to the data of young mice as 1. Data are shown as mean ± SEM [*n* = 4 mice; *p < 0.05, **p < 0.01, ***p < 0.001 by Student’s *t* test (two-tailed)]. **b** Photos of the cross-section of myofibers. Aged C57BL/6J mice (~24-month-old) were injected with miR199#4 (199#4) or nsCont to the TA muscle, and 2 days later the TA muscles were examined as in Fig. 4b. Scale bars indicate 100 µm. **c** Averaged cross-sectional area of myofibers. The cross-sectional areas (CSAs) of myofibers in the TA muscle were measured and analyzed as in Fig. 4d. The CSAs of myofibers in untreated aged mice (−) were also examined. The number of mice examined was as follows: six miR199#4-dosed mice, three nsCont-dosed mice, and four untreated mice. Data are shown as mean ± SEM [**p < 0.01, ***p < 0.001 by ANOVA (Tukey’s post hoc test)]. **d** Histogram of the CSAs of myofibers. Histogram of the CSAs of myofibers was analyzed and displayed as in Fig. 4e. **e**, **f** Analyses of the CSAs of myofibers in aged mice intravenously injected with miR199#4. Aged C57BL/6J mice (~24-month-old) were intravenously injected with miR199#4 or nsCont from the tail vein. One week later, the CSAs of myofibers in the TA muscle were examined as in Fig. 4b; and averaged CSA (**e**) and histogram of the CSAs (**f**) were analyzed as in Fig. 4d, e, respectively. The data of averaged CSAs (**e**) are shown as mean ± SEM [*n* = 4 treated mice; *p < 0.05 by Student’s *t* test (two-tailed)].

We examined the effects of miR199#4 on aged mice (~24-month-old). Aged mice exhibited a significant decrease in muscle miR-199-3p (Fig. 5a), as in blood-circulating cf-miR-199-3p, compared to young mice. We administered miR199#4 to the TA muscles of aged mice, and 2 days later the TA muscles were examined by immunohistochemical analysis (Fig. 5b). The mean cross-sectional area of myofibers treated with miR199#4 was significantly larger than that of control myofibers treated with nsCont (Fig. 5c). Consistent with this, the size-divided histogram analysis revealed a marked increase in enlarged myofibers in the presence of miR199#4 (Fig. 5d).

Since blood-circulating cf-miR-199-3p is reduced in aged mice, we introduced miR199#4 into the circulating blood of aged mice via the tail vain. After intravenous administration of miR199#4, the cross-sectional area of myofibers was examined and analyzed, as described above. The results, as shown in Fig. 5e, f, are consistent with the results of the local administration of miR199#4 to aged muscles (Fig. 5c, d).

**Fig. 6:**
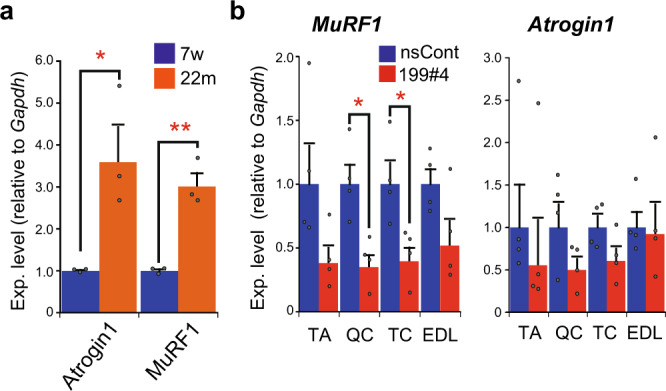
Suppression of genes related to the muscle atrophy pathway by miR199#4 treatment. **a** Expression of *Atrogin1* and *MuRF1* in young and aged muscles. The expression of *Atrogin1* and *MuRF1*, which are involved in the muscle atrophy pathway, in young (7-week-old: 7w) and aged (22-month-old: 22m) mice was examined by qRT-PCR as in Fig. 1b, and normalized to the data of young mice as 1. Data are shown as mean ± SEM [*n* = 3 mice; *p < 0.05, **p < 0.01 by Student’s *t* test (two-tailed)]. **b** Suppression of *MuRF1* and *Atrogin1* by miR199#4 treatment. Total RNA was extracted from the quadriceps (QC), triceps (TC), extensor digitorum longus (EDL), and TA muscles in aged mice intravenously injected with miR199#4 (199#4) or nsCont (the same mice examined in Fig. 5e, f), and the level of *MuRF1* and *Atrogin1* was examined by qRT-PCR followed by the delta–delta Ct analysis as in Fig. 2a, b. Data are shown as mean ± SEM [*n* = 4 treated mice; *p < 0.05 by Student’s *t* test (two-tailed)].

It should be noted that there is a difference in enlarged muscle fibers between the muscle injury model and aged mice. Central nuclei are seen in regenerating muscle fibers (Fig. 4b), but such myofibers were hardly detected in aged mice treated with miR199#4 (Fig. 5b). This suggests that preexisting muscle fibers may become enlarged in aged mice following miR199#4 treatment. We hypothesized that another mechanism different from muscle regeneration might be involved in myofiber enlargement in aged mice, and proposed the involvement of the muscle atrophy pathway. The *Atrogin1* and *MuRF1* genes are closely related to muscle atrophy^38^, and their expression levels are significantly elevated in aged muscles compared to young muscles (Fig. 6a). We examined the level of *Atrogin1* and *MuRF1* in muscles of aged mice after systemic administration of miR199#4. *Atrogin1* and *MuRF1* were markedly reduced in various muscles by miR199#4 administration (Fig. 6b), suggesting that suppression of the muscle atrophy pathway by miR199#4 may cause myofiber expansion in aged mice.

### MiR199#4 delays loss of muscle strength in aged mice

**Fig. 7:**
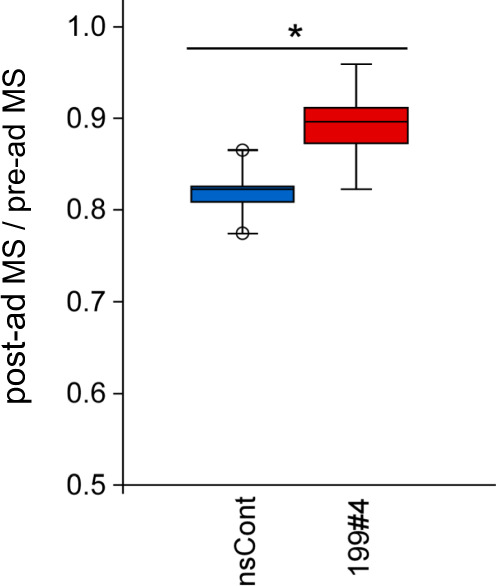
Grip strength test of aged mice. Eighty-week-old (20-month-old) C57BL/6J mice were subjected to the grip strength test, and 1 week later, miR199#4 (199#4) and nsCont were intravenously administered. The grip strength test was carried out again 9 weeks later. The data were normalized by body weight, and the ratio of post-administered muscle strength (post-ad MS) to pre-administered muscle strength (pre-ad MS) was calculated. Data are shown as mean ± SEM [*n* = 5 mice; *p < 0.05 by Student’s *t* test (two-tailed)].

The effect of miR199#4 on muscle function in aged mice is of interest. The grip strength test was performed on aged mice before and after systemic administration of miR199#4. The experiment was performed in a double-blind manner; RNA injection and the grip test were carried out by different experimenters without sharing information. C57BL/6J male mice (80-week-old) were grip-tested, then 1 week later, they were injected intravenously with miR199#4 and nsCont, and grip-tested again. The change in muscle strength after miR199#4 injection was examined. Age-related muscle weakness in miR199#4-treated mice was significantly suppressed compared to that in control mice treated with nsCont (Fig. 7).

### MiR199#4 improves the muscle strength of mdx mice

**Fig. 8:**
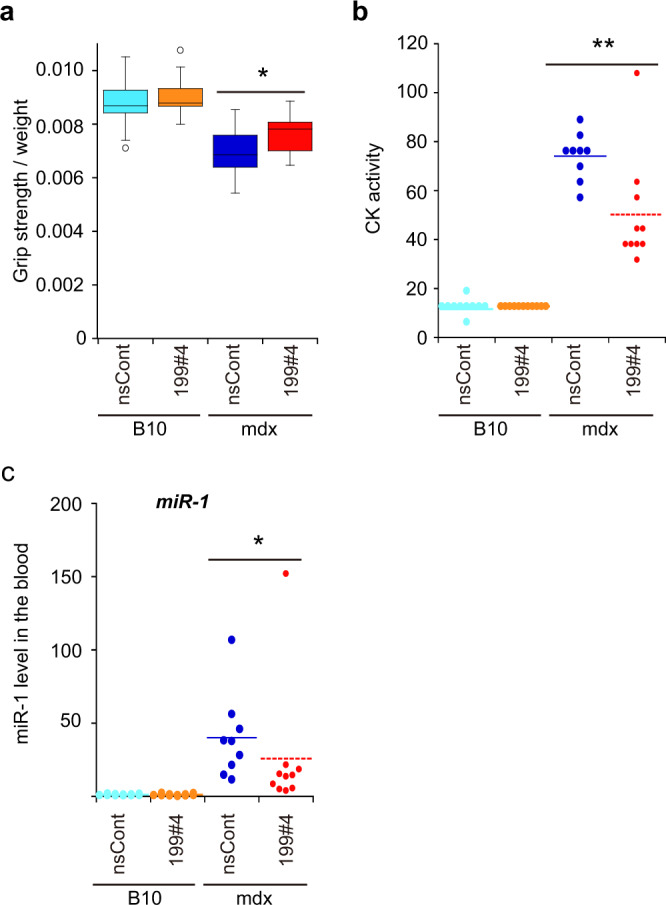
Effects of miR199#4 on mdx mice. **a** Grip strength test of mdx mice. Mdx and B10 control mice (8-week-old) were injected twice intravenously with miR199#4 (199#4) and nsCont, and examined by the grip strength test 1 week after the last administration. The first two grip strength data were normalized by body weight and averaged. The number of mice examined was as follows: 13 miR199#4-dosed mdx mice, 12 nsCont-dosed mdx mice, 11 miR199#4-dosed B10 mice, and 13 nsCont-dosed B10 mice. Data are shown as mean ± SEM [*p < 0.05 by Student’s *t* test (two-tailed)]. **b** Creatine kinase (CK) activity. 1 week after the first administration, blood samples were collected and serum CK activity was measured. Data are plotted and shown in an arbitrary unit. Meanscore is indicated by a dotted bar [**p < 0.01 by Wilcoxon rank-sum test]. **c** Cf-miR-1 in the blood. The level of cf-miR-1 was examined in the same blood samples as **b** by qRT-PCR and analyzed as in Fig. 1a. The data were normalized with the averaged data of nsCont-dosed B10 mice as 1. Data are plotted, and meanscore is indicated by a dotted bar (*p < 0.05 by Wilcoxon rank-sum test). The number of mice examined in **b** and **c** was as follows: 10 miR199#4-dosed mdx mice, 9 nsCont-dosed mdx mice, 11 miR199#4-dosed B10 mice, and 11 nsCont-dosed B10 mice.

Since miR199#4 has a positive impact on muscles, we conducted an experiment to assess the effect of miR199#4 administration in mdx mice, a well-known DMD animal model^32^. Eight-week-old mdx mice were injected intravenously with miR199#4 (1.6 mg/kg) twice (1 week interval), and the grip test was carried out 1 week after the last administration. The experiment was performed in a double-blind manner as shown in Fig. 7. The results (Fig. 8a) show a significant improvement in muscle strength in mdx mice treated with miR199#4 compared to mdx mice treated with nsCont. In addition, significant decreases in creatine kinase (CK) activity and cell-free miR-1 were observed in the blood of mdx mice treated with miR199#4 (Fig. 8b, c), which are possible signs of the disease improvement.

As a drug delivery reagent, atelocollagen is viscous and difficult to handle, and the mice treated with atelocollagen appeared to suffer negative effects due to the vehicle. Thus, we looked for alternatives to atelocollagen to improve our drug delivery system (DDS) and found DC-CHOL/DOPE cationic liposomes as a new DDS reagent that is equal or superior to atelocollagen (Supplementary Fig. 4). The drug distribution of miR199#4 with the cationic liposomes showed that the administered miR199#4 was delivered to the muscle and consistently taken up in large amounts in the liver (Supplementary Fig. 5). Extracellular miR199#4 delivered to the muscle may function as a mediator in gene silencing and contribute to the gene regulation involved in the improvement of muscle strength.

We attempted to identify biomolecules related to the improvement following miR199#4 administration. Various polypeptides related to DMD were examined by western blotting; however, distinct signals related to the improvement were not detected in this study (Supplementary Fig. 6). We will discuss this point further in the following section.

## Discussion

Biomolecules change qualitatively and quantitatively with aging^1–3^. Capturing such changes and studying them are vital for understanding aging, and may reveal strategies for dealing with problems in aging societies. Parabiosis studies in which young and aged animals are conjoined to share a common vascular system have provided important insights into aging and rejuvenation^9–12^. The findings strongly suggest that there are rejuvenating and aging factors in young and aged circulating blood, respectively. Previous studies reported various molecules as candidates related to rejuvenation and aging, but the results are controversial^13^.

Our in vitro cell culture experiments using young and aged sera are similar to parabiosis experiments (Fig. 1b), and we found miR-199-3p, which changes with age, in circulating blood; the miRNA was identified to significantly decrease in aged blood (Table 1 and Fig. 1a). In addition, the expression of muscle (cellular) miR-199-3p markedly decreases in aged muscles compared to young muscles (Fig. 5a); thus, cf-miR-199-3p in the blood may correlate with muscle miR-199-3p. The decrease in expression of miR-199-3p with aging is also observed in mesenchymal stem cells derived from rhesus macaque bone marrow^39^. Therefore, the expression of cellular miR-199-3p may be under age-related control. In contrast to miR-199-3p, the expression of cellular myogenic miRNAs, such as miR-1 and miR-133, remain unchanged in young and aged muscles (Fig. 5a), but the level of their extracellular miRNAs in the blood is markedly reduced in aged mice (Fig. 1a). The difference between miR-199-3p and myogenic miRNAs (miR-1 and -133) may reflect the difference in cf-miRNA processing and/or in age-related control of cf-miRNAs between them. To elucidate this difference, further studies need to be conducted in the future.

Previous studies suggested that miR-199-3p participates in various cellular functions and vital phenomena via suppression of its target genes; e.g., the miRNA may promote the proliferation and migration of endothelial tip cells and modulate the onset of puberty, respectively, through downregulation of the targeted semaphorin-3A gene^40^ and through suppression of target genes (*Cdc42*, *Map3k2*, *Map3k4*, and *Taok1*) involved in the p38 MAPK pathway^41^. The present study has found that miR-199-3p is involved in myogenic differentiation through suppression of its target genes, *Lin28b* and *Suz12* (Fig. 3). Lin28b is an RNA-binding protein and inhibits the maturation of miRNAs, including miR-1, which is a muscle-specific miRNA and promotes myogenesis^37^. Suppression of Lin28b by miR-199-3p causes an increase in matured miR-1 (Fig. 3), which may allow myogenic differentiation to proceed.

Suz12 is a core subunit of polycomb repressive complex 2 (PRC2), which participates in repressing chromatin^42^. Gene regulation of PRC2 is involved in stem cell maintenance, embryonic development, cell proliferation and differentiation, including myogenic differentiation^42^. PRC2 is present at silent muscle-specific loci, such as *Myog* and *muscle creatine kinase* (*MCK*) in undifferentiated myoblasts, but is dissociated from such loci in differentiated myotubes^43^. Gene silencing against *Suz12* by RNAi causes the enhancement of myogenic gene expression (Fig. 3). Suppression of the enhancer of the zeste homolog 2 (Ezh2) protein, another core subunit of PRC2, also causes myogenic differentiation^44^. These findings suggest that inhibition of PRC2 formation due to suppression of its core subunits may trigger the creation of a genomic state for muscle differentiation. Thus, suppression of Suz12 by miR-199-3p may induce myogenic differentiation. Taken together, miR-199-3p may play an important role in myogenic differentiation through inhibition of two independent pathways involving Lin28b and Suz12, which are each involved in the maintenance of the undifferentiated state of myoblasts.

The effect of miR-199-3p on myogenic differentiation has also been shown in vivo (Fig. 4). Muscle damage triggers cell proliferation and differentiation due to muscle regeneration. Introduction of miR199#4, which supplies miR-199-3p, to damaged sites enhanced muscle regeneration and expanded regenerating myofibers (Fig. 4). Myogenic gene expression was consistently upregulated under miR199#4 treatment (Fig. 4). From a practical viewpoint, miR199#4 might be effective and useful for surgical treatments.

When miR199#4 was administered to aged mice lacking blood-circulating miR-199-3p, the mice exhibited markedly expanded myofibers (Fig. 5) and delayed loss of muscle strength with aging (Fig. 7). The expanded myofibers in aged mice differ from regenerating myofibers, which are enlarged in the presence of miR199#4; the former is derived from preexisting myofibers, and the latter is from neo-myofibers (newborn myofibers). Thus, there are different, independent mechanisms in the muscle fiber enlargements. Our study suggests that (i) in the former case, inhibition of muscle atrophy due to suppression of *Atrogin1* and *MuRF1* by miR199#4 treatment brings about enlargement of aged myofibers, and (ii) in the latter case, suppression of target genes, *Lin28b* and *Suz12*, causes enlargement of regenerating myofibers. Altogether, different (multiple) genes regulated by miR-199-3p may be involved in muscle hypertrophy in different situations. The findings also suggest that miR-199-3p may have at least an antiaging effect on muscle, and that miR199#4 supplying miR-199-3p may have potential as a novel RNA medicine for age-related muscle diseases, such as sarcopenia.

Based on the high efficacy of miR199#4 on muscles, we administered miR199#4 to mdx mice, a well-known DMD model, and obtained remarkable results: systemic administration of miR199#4 improved muscle strength in the mice by ~20% (Fig. 8a). To understand the mechanism of the muscle strength recovery, we attempted to identify biomolecules that were improved (or influenced) by miR-199-3p. However, with the exception of improvements of blood CK and cf-miR-1 (Fig. 8b, c), we were unable to detect such improvements at the molecular level. This difficulty in detection may be due to the complex cell population, which consists of a mix of regenerating and disrupting muscle cells in mdx mice. Even if miR199#4 results in the improvement, the high background noise caused by the complex cell population would mask evidence of the improvement. To solve this problem, more extensive studies using a homogenous muscle cell population derived from mdx mice or focusing on various tissues, including muscle need to be carried out.

Although the molecular mechanism of the muscle strength recovery by miR199#4 remains unknown, the dose of miR199#4 for improved muscle strength is noteworthy. Two times administration of ~1.6 mg/kg miR199#4 improved muscle strength in mdx mice. The effective dose appears to be lower than that of antisense oligonucleotides for exon-skipping against mutant dystrophin genes in vivo^45–47^. The reason why miR199#4 is capable of improving muscle strength at such lower doses may be due to its different mechanism of action compared with antisense oligonucleotides. The mechanism of action of miR199#4 is catalytic gene silencing. In contrast, the mechanism of antisense oligonucleotides is based on physical hybridization. Therefore, differences in the appropriate doses to show drug efficacy may be attributable to the different mechanisms of action.

The mechanism by which organs, tissues, or cells release miRNAs into the circulating blood is not yet fully understood, and the age-related regulation on the release of such miRNAs is also unclear. The present study showed that myogenic cf-miRNAs, including cf-miR-199-3p circulate with the blood in the vascular system and decrease with aging. Such cf-miRNAs may be present in exosomes, i.e., they are exosomal miRNAs.

Myogenic miRNAs participate in the myogenic differentiation, muscle regeneration, and maintenance of muscular homeostasis^33^. Previous studies reported that myogenic miRNAs, such as miR-1 and miR-133, were observed to increase in the blood in response to physical exercise^48,49^. Based on these reports, active young mice may release more myogenic miRNAs into the blood than aged slow-moving mice, and such extracellular miRNAs, including cf-miR-199-3p may contribute to maintaining muscle homeostasis in young mice through autocrine action. However, the level of extracellular (exosomal) miRNA does not always correlate with the cellular miRNA level, and differences have been observed in myogenic miRNAs and miR-199-3p between young and aged mice (Figs. 1a and 5a) and during myogenic differentiation (Supplementary Fig. 2a). Thus, as another possibility, it is conceivable that age-related and differentiation-related regulation of exosome formation may affect cf-miRNA production; specifically, there may be differences in exosome formation between young and aged mice. In the context of these hypotheses, further study is needed to elucidate the age-related release of myogenic miRNAs into the blood.

Aside from the release mechanism, when young myogenic cf-miRNAs containing cf-miR-199-3p flow into the aged blood-circulating system from a young vascular system by parabiosis, the cf-miRNAs could trigger muscle activation and inhibition of muscle atrophy on the aged side; this concept is supported by evidence from aged mice intravenously injected with miR199#4 in this study (Figs. 5–7). Cf-miRNAs in the blood may be related to age-related homeostatic function and could represent altered homeostasis. When such cf-miRNAs are ectopically present, for example, when cf-miRNAs in young blood are transferred to the aged blood-circulating system, some cf-miRNAs may show rejuvenating effects on the aged side, i.e., they may produce antiaging effects. In contrast, cf-miRNAs in aged blood may represent altered homeostasis with aging. When such cf-miRNAs are transferred to the young blood-circulating system, some cf-miRNAs might trigger aging-related deterioration and might cause premature aging on the young side. Cf-miRNAs that are significantly increased in aged blood represent candidates that will require detailed mechanistic analysis to understand and prevent aging.

In conclusion, our current study has provided new insights into cf-miRNAs as age-related molecules, and revealed a new research direction to clarify the relationship between aging and cell-free functional RNAs, including cf-miRNAs.

## Methods

### Mice

C57BL/6J male mice were purchased from Charles River Laboratories Japan, Inc. (Yokohama, Kanagawa, Japan) or CLEA Japan, Inc. (Tokyo, Japan). Mdx and B10 mice were purchased from Central Institute for Experimental Animals (Kawasaki, Kanagawa, Japan). Mice were housed, fed, and maintained in the laboratory animal facility according to the National Institute of Neuroscience animal care guidelines. All the animal experiments were performed in strict accordance with the recommendations in the Guide for the Care and Use of Laboratory Animals of the National Institutes of Neuroscience. The protocols were approved by the Committee on the Ethics of Animal Experiments of the National Institutes of Neuroscience (Permit Number: 20188005).

### Cell culture

C2C12 cells, a mouse myoblast cell line, were obtained from RIKEN BRC Cell Bank and grown in Dulbecco’s modified Eagle’s medium (DMEM; FUJIFILM Wako Pure Chemical Corp., Osaka, Japan) supplemented with 10% fetal bovine serum (Thermo Fisher Scientific, Waltham, MA, USA), 100 units/ml penicillin, and 100 μg/ml streptomycin (FUJIFILM Wako) at 37 °C in a 5% CO_2_ humidified chamber. Myogenic differentiation was performed at 30 °C by replacing the growth medium with differentiation medium consisting of DMEM supplemented with 2% horse serum^50^, and the differentiation medium was changed every other day. Neuro2a cells, a mouse neuroblastoma cell line, were cultured as described previously^19^.

### RNA and DNA oligonucleotides

RNA and DNA oligonucleotides used in this study were synthesized by and purchased from Sigma-Aldrich Japan (Tokyo, Japan). The sequences of synthesized oligonucleotides were shown in Supplementary Tables 1 and 2. The MISSON^®^ microRNA mimics and miRCURY LNA miRNA Inhibitors were purchased from Sigma-Aldrich Japan and QIAGEN (Hilden, Germany), respectively.

### Collection of whole blood and preparation of plasma, serum, and exosomes

Whole blood was collected from mice by cardiac puncture using a syringe containing 0.1 ml of 10 mg/ml EDTA with a 26 G needle. The collected blood samples were centrifuged at 1200 × *g* for 15 min at room temperature, followed by at 2000 × *g* for 20 min at room temperature, and the supernatant was collected as a plasma sample. For preparation of blood serum, whole blood was collected in the absence of EDTA and incubated at room temperature for 30 min, followed by centrifugation at 1200 × *g* for 15 min at room temperature. Resulting supernatant was collected as a serum sample and stored at −80 °C. The mouse serum was heat inactivated at 56 °C for 30 min, and filtered through a 0.2 µm filter prior to cell culture.

For isolation of exosomes, plasma samples were further centrifuged at 12,000 × *g* for 45 min at room temperature, and 200 μl of the plasma sample mixed with 100 μl of PBS and 60 μl of a Total Exosome Isolation (for plasma; Thermo Fisher Scientific). The mixture was incubated at room temperature for 10 min, and centrifuged at 10,000 × *g* for 30 min at 4 °C to collect exosomes. The collected exosomes were suspended in 200 μl of PBS.

### Total cellular RNA and cell-free RNA isolation

Total RNA was extracted from cells and tissues by a TRI Reagent (MRC, Cincinnati, OH, USA) according to the manufacturer’s instructions.

Cell-free RNAs were isolated from plasma and exosomal samples using a Plasma/Serum Circulating and Exosomal RNA Purification Mini Kit (Slurry Format; NORGEN, Thorold, ON L2V 4Y6 Canada), according to the manufacturer’s instructions. For normalization, 5 pg of Syn-cel-miR-39-3p miScript miRNA Mimic (QIAGEN) was added as an external control to each lysis sample.

### Muscle injury and administration of miRNA

Muscle injury was performed by intramuscular injection of 50 µl of 1.2% (w/v) BaCl_2_ (in saline) into the TA in left hind limbs of mice (8-week-old). One day after muscle injury, synthetic miRNAs (miR199#4 and nsCont) prepared with atelocollagen as a drug delivery reagent were intramuscularly administered to the damaged sites. Briefly, 5 μM miRNA–atelocollagen mixture was prepared using an AteloGene® Local Use “Quick Gelatin” kit (KOKEN, Tokyo, Japan), according to the manufacturer’s instructions, and 50 μl of the mixture was injected to the damaged TA. Ten days after muscle injury, treated TA was examined.

### Systemic administration of miRNAs

For systemic administration of synthetic miRNAs, 15 μM miRNA–atelocollagen mixture was prepared using an AteloGene Systemic Use kit (KOKEN), according to the manufacturer’s instructions, and 200 μl of the mixture was intravenously administered per mouse from the tail vein. As an alternative method, DC-CHOL/DOPE cationic liposomes (FormuMax Scientific Inc, Sunnyvale, CA, USA) were used. A total of 15 μM miRNA-DC-CHOL/DOPE complexes were prepared in 200 μl PBS and intravenously administered from the tail vein per mouse.

### Grip strength test

The conventional forelimb grip strength test was performed by an experimenter who did not know the treatment information of test mice, using a Grip Strength Mater (GPM-100; Melquest, Toyama, Japan). Briefly, a mouse was allowed to grasp the bar mounted on the grip strength meter. After stabilization, the gauge of the meter was rested to 0 g, and the mouse tail was slowly pulled by the experimenter^51,52^. Tension was recorded by the gauge at the time when the mouse released its forepaws from the bar. The test was consecutively carried out five times with ~5 s intervals. The data were normalized by the body weight of test mouse and averaged. Normalized data were analyzed using two-way analysis of variance (ANOVA) followed by a post hoc comparison test.

### Immunohistochemical staining and analysis

TA muscles were isolated from mice, washed in saline, and immediately frozen in isopentane chilled in liquid nitrogen. The frozen muscles were sliced into 10 µm thick cryosections. The cryosections were placed on slides and fixed in chilled acetone (−20 °C) for 10 min, followed by washing in PBS twice. The fixed cryosections were blocked with blocking buffer (5% goat serum and 1% BSA in PBS) for 15 min at room temperature, and treated with a diluted primary antibody [1/200 anti-Laminin-α2 (ENZO, Farmingdale, NY, USA)] in blocking buffer at 4 °C overnight, followed by incubation with 1:500 diluted Goat Anti-Rat Alexa 488 (Thermo Fisher Scientific) as a secondary antibody at room temperature for 2 h. After washing and staining with DAPI, the processed cryosections were examined by a fluorescent microscope [Axiovert 40 CFL (Carl Zeiss, Jena, Germany) or BZ-X700 (KEYENCE, Osaka, Japan)]. Cross-section area of each myofiber was measured by the ImageJ software.

### Electroporation

Electroporation was carried out to introduce synthetic miRNA mimics or inhibitors (90 pmol/test) to C2C12 cells by a Nucleofector™ 2b (Lonza, Basel, Switzerland) with an Amaxa Cell Line Nucleofector Kit V (Lonza), according to the manufacturer’s instructions. After electroporation, the cells were cultured in differentiation medium at 30 °C. MiRNA mimics purchased from Sigma-Aldrich are as follows (catalog numbers are indicated in parentheses):

miR-1 (HMI0046), miR-133a (HMI0196), miR-133b (HMI0198), miR-26a (HMI0415), miR-125b (HMI0112), miR-199a-3p (HMI0338), miR-214 (HMI0379), let-7e (HMI0013), and negative control 1 (HMC0002).

Inhibitors purchased from QIAGEN are as follows:

miR-199a-3p inhibitor (YI04100433) and inhibitor control (negative control A; YI00199006).

### Construction of reporter plasmids

OligoDNA duplexes encoding the predicted miR-199a-3p target sequences in the *Lin28b* and *Suz12* 3′UTRs, and carrying the miR-199a-3p and its complimentary sequences were chemically synthesized (Supplementary Table 2), and inserted into the 3′UTR of *Renilla luciferase* in the psiCHECK-2 vector (Promega), as described previously^53,54^.

### Transfection and reporter assay

The day before transfection, cells were trypsinized and seeded onto 96-well culture plates (0.5 × 10^4^ cells/well). The constructed psiCHECK-2-backbone plasmids (20 ng/well) and synthetic miRNA mimics (20 nM final concentration) were co-transfected into cells by using a Lipofectamine2000 transfection reagent (Thermo Fisher Scientific), according to the manufacturer’s instructions. After 24 h, cells were washed with PBS and lysed in 1× passive lysis buffer (Promega), and the expression of *Photinus* and *Renilla* luciferases was examined by a Dual-Luciferase Reporter Assay system (Promega), according to the manufacturer’s instructions. The luminescent signals were measured by a Synergy H1 Multi-Mode Reader (BioTek, Winooski, VT, USA).

### Quantitative reverse transcription-polymerase chain reaction

Complementary DNA (cDNA) synthesis for miRNAs was carried out using a TaqMan MicroRNA Reverse Transcription Kit (Thermo Fisher Scientific) with TaqMan MicroRNA Assays (Thermo Fisher Scientific) by a SimpliAmp Thermal Cycler (Thermo Fisher Scientific), according to the manufacturer’s instructions. Subsequently, quantitative PCR was performed using a StepOne Plus Real-Time PCR system (Thermo Fisher Scientific) with a TaqMan® Fast Advanced Master Mix (Thermo Fisher Scientific) together with TaqMan MicroRNA Assays (Thermo Fisher Scientific), according to the manufacturer’s instructions. TaqMan MicroRNA Assays used are as follows (assay IDs are indicated in parentheses):

mmu-miR-1a-3p (2222), mmu-miR-133a-3p (2246), mmu-miR-133b-3p (2247), mmu-miR-214-3p (2306), mmu-miR-199a-3p (2304), mmu-miR-26a-5p (405), mmu-miR-125b (449), mmu-let-7e-5p (2406), mmu-miR-6412 (474230_mat), mmu-miR-351-5p (1067), mmu-miR-3474 (243093_mat), mmu-miR-24-3p (402), U6 snRNA (1973), and cel-miR-39 (200).

To examine endogenous gene expression, total RNAs were subjected to cDNA synthesis using oligo (dT)_15_ primers (Promega) and a SuperScript® III reverse transcriptase (Thermo Fisher Scientific), according to the manufacturer’s instructions. The resulting cDNAs were subjected to quantitative PCR analysis using a StepOne Plus Real-Time PCR system (Thermo Fisher Scientific) with a Fast SYBR® Green Master Mix (Thermo Fisher Scientific) and Perfect Real-Time primers (TAKARA BIO, Kusatsu, Shiga, Japan), according to the manufacturer’s instructions. The Perfect Real-Time primers used are as follows (TAKARA BIO primer set IDs):

*Myog* (MA127738), *Myh1* (MA149010), *Myod1* (MA128901), *Gapdh* (MA050371), *Myf5* (MA075089), *Ckm* (MA112761), *MuRF1* (*Trim63*; MA056880), *Atrogin1* (*Fbxo32*; MA173861), and *Actb* (MA050368).

### Western blot

Tissue samples were lysed with cell lysis buffer (20 mM Tris-HCl, pH 7.5, 150 mM NaCl, 1 mM EGTA, and 1% Triton X-100) containing 1× protease inhibitor cocktail (Protease Inhibitor Cocktail Tablets; Roche Diagnostics, Basel, Switzerland) and homogenized with a pestle, followed by three times pulse sonication (5 s sonication at a 100% amplitude and 10 s resting interval) on ice, using a QSONICA Q125 (WAKEN B TECH, Tokyo, Japan). The lysate was centrifuged at 14,000 × *g* for 15 min at 4 °C, and the supernatant was collected as a protein sample. In the case of cultured cells, cells were washed with PBS (Wako) and lysed in RIPA buffer (Thermo Fisher Scientific) containing 1× protease inhibitor cocktail (Cell Signaling Technology, Danvers, MA, USA) on ice for 5 min. The lysate was passed through a 26 G needle with a 1 ml syringe ten times and centrifuged at 14,000 × *g* for 15 min at 4 °C. The supernatant was collected as a protein sample. Protein concentration of the samples was measured by a protein quantification kit-wide range (DOJINDO, Kumamoto, Japan). Equal amounts of protein were mixed with 2× sample buffer (125 mM Tris-HCl, pH6.8, 2% glycerol, 4% SDS, 0.02% bromophenol blue, and 10% β-mercaptoethanol) and boiled for 5 min. The protein samples were electrophoretically separated on 8% or 10% SDS–polyacrylamide gels, and blotted onto polyvinylidene fluoride membranes (Immobilon P; Millipore, Billerica, MA, USA). The membranes were incubated in blocking buffer (5% skim milk in TBS-T buffer [Tris-buffered saline (TBS) containing 0.1% Tween20]) for 1 h and incubated with diluted primary antibodies at 4 °C overnight. After incubation, the membranes were washed in TBS-T buffer and incubated with 1/5000 diluted horseradish peroxidase-conjugated goat anti-mouse IgG (Sigma-Aldrich) or goat anti-rabbit IgG (Sigma-Aldrich) for 30 min at room temperature. Antigen–antibody complexes were visualized using an ECL Prime Western Blotting Detection Reagent (GE healthcare, Chicago, IL, USA), according to the manufacturer’s instructions. The original blot images used in figures are shown in Supplementary Fig. 7. The primary antibodies used are indicated below with the product IDs and dilution ratios in parentheses:

Anti-Suz12 (#3737, 1/1000), anti-Lin28b (#5422, 1/1000), and anti-Gapdh (#2118, 1/5000) were purchased from Cell Signaling Technology. Anti-Myosin heavy chain (MF20; MAB4470, 1/200) and anti-α-tubulin (F2168, 1/5000) were purchased from R&D systems (Minneapolis, MN, USA) and Sigma-Aldrich, respectively. Anti-dystrophin (ab15277, 1/1000), anti-α-sarcoglycan (ab189254, 1/1000), and anti-dysferlin (ab124684, 1/1000) were purchased from Abcam (Cambridge, UK). Anti-utrophin (610896, 1/1000) and anti-β-dystyroglycan (849401, 1/500) were purchased from BD Transduction Laboratories (Franklin Lakes, NJ, USA) and BioLegend (San Diego, CA, USA), respectively.

### Enzyme-linked immuno-sorbent assay (ELISA) and creatine kinase activity assay

The levels of IGF-I and CK in mouse serum were quantified with a Mouse/Rat IGF-I Quantikine ELISA kit (MG100, R&D systems) and a Creatine Kinase Activity Assay Kit (Clorimetric; ab155901, Abcam), respectively, according to the manufacturers’ instructions.

### DNA chip analysis

3D-Gene DNA chips (Mouse miRNA ver.19; TORAY, Tokyo, Japan) were used for detection of mouse miRNAs.

### Accession number

The Gene Expression Omnibus (GEO) accession number of the miRNA expression data used in this study is GSE141258.

### Statistics and reproducibility

Data obtained in this study were initially evaluated by one-way ANOVA. If significant difference between data was detected by ANOVA, Tukey’s post hoc test, or Dunnett’s test was carried out between the data of interest. The level of statistical significance was set at 0.05.

### Reporting summary

Further information on research design is available in the Nature Research Reporting Summary linked to this article.

## Supplementary information

Supplementary Information

Description of Additional Supplementary Files

Supplementary Data 1

Supplementary Data 2

Reporting Summary

## Data Availability

The datasets generated during and/or analyzed during the current study are available in the Gene Expression Omnibus (GEO) repository (accession number: GSE141258) in the NCBI. All data generated or analyzed during this study are included in this published article (and its Supplementary Information files), and the source data underlying the graphs are provided in Supplementary Data 1 and 2.
