# Supplementary material for: MiR-199-3p enhances muscle regeneration and ameliorates aged muscle and muscular dystrophy
Source: Commun Biol. 2021 Mar 29;4:427. doi: 10.1038/s42003-021-01952-2 (PMC8007565; doi:10.1038/s42003-021-01952-2)
Supplement: Supplementary file 1 — Supplementary Information [file 42003_2021_1952_MOESM1_ESM.pdf]

MiR-199-3p enhances muscle regeneration and ameliorates aged muscle and muscular dystrophy

Masashi Fukuoka[1,!], Hiromi Fujita[2], Kosumo Numao[1], Yasuko Nakamura[2], Hideo Shimizu[1], Masayuki Sekiguchi[2], Hirohiko Hohjoh[1,*]

[1]Department of Molecular Pharmacology, National Institute of Neuroscience, NCNP, Tokyo, Japan
[2]Department of Degenerative Neurological Diseases, National Institute of Neuroscience, NCNP, Tokyo, Japan

! Present address:
Department of Biological Science and Technology, Faculty of Industrial Science and Technology, Tokyo University of Science, Tokyo, Japan

***Corresponding Author**: Hirohiko Hohjoh, Ph.D.
Department of Molecular Pharmacology, National Institute of Neuroscience, NCNP 4-1-1 Ogawahigashi, Kodaira, Tokyo 187-8502, JAPAN
Tel: +81-42-342-2711, ext. 5234, 5951, Fax: +81-42-346-3594
E-mail: hohjohh@ncnp.go.jp

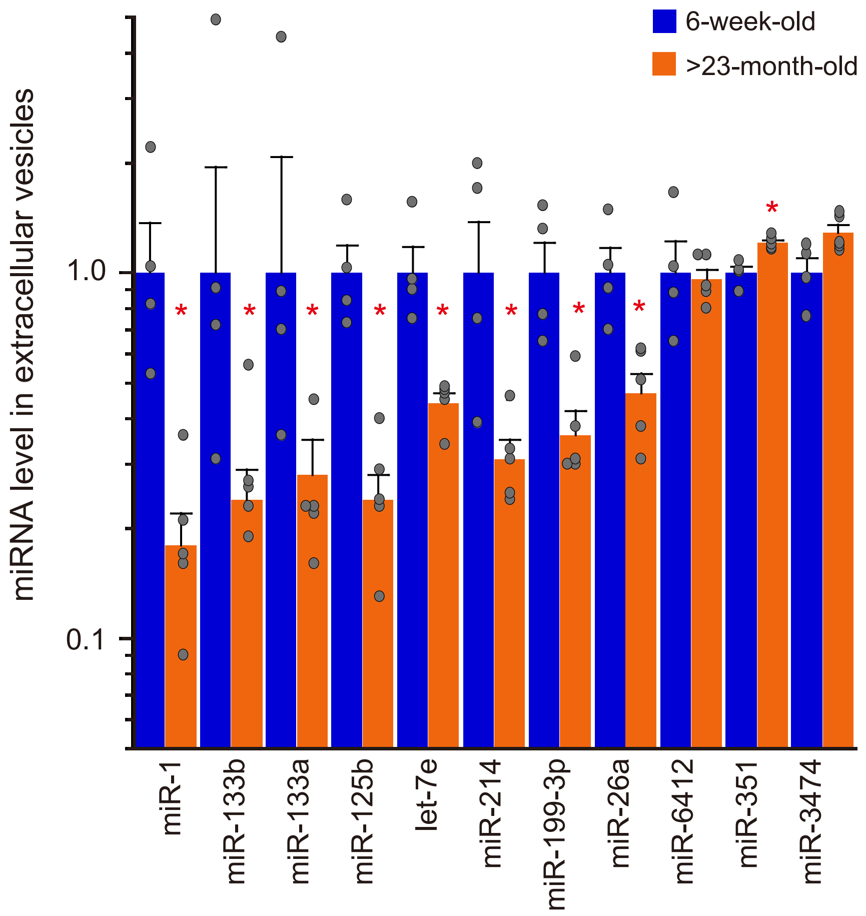

Supplementary Fig. 1. Exosomal miRNAs in young and aged mouse blood. Extracellular vesicles containing exosomes were prepared from the plasma of young (6-week-old) and aged (>23-month-old) C57BL/6J mouse blood. RNA in the vesicles was extracted and examined by qRT-PCR followed by the delta-delta Ct method as in Fig. 1a. Examined miRNAs are indicated. Data are shown as mean±SEM (n = 5 mice; *p<0.05)

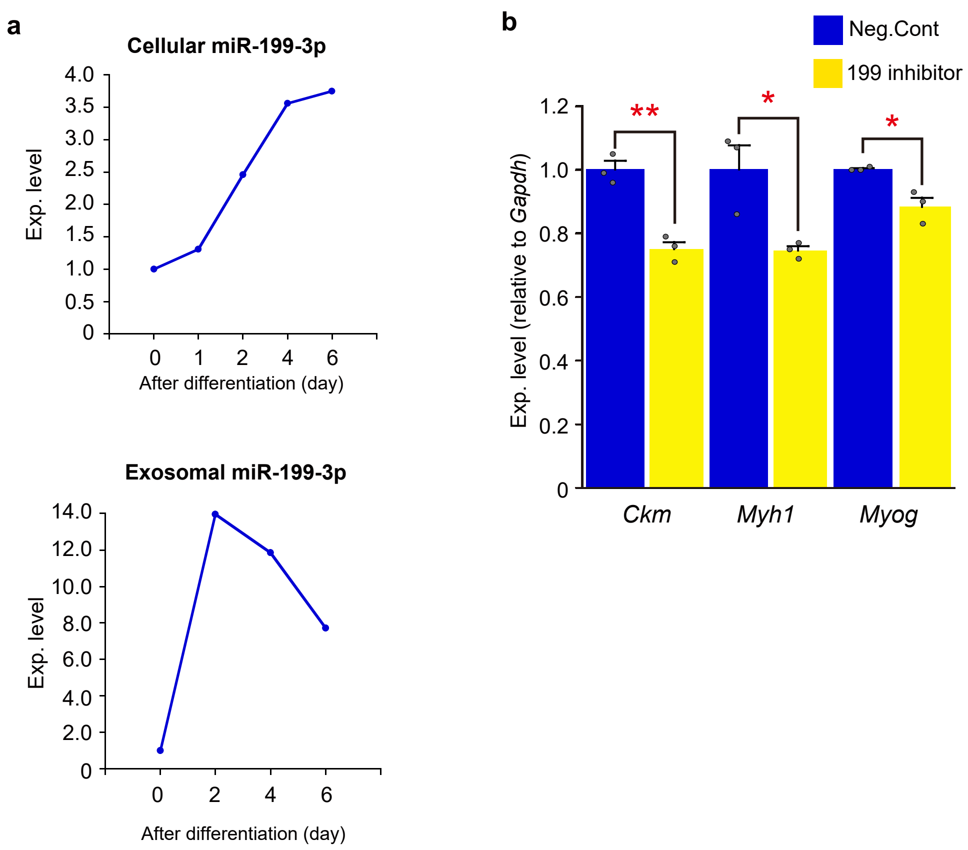

Supplementary Fig. 2. (a) Cellular and exosomal miR-199-3p during the differentiation of C2C12 cells. C2C12 cells were cultured in differentiation medium containing 2% horse serum at 30°C in a 5% CO2 humidified chamber. Total RNA and exosomal RNA were extracted from cells and extracellular vesicles (prepared from conditioned media), respectively, at the indicated days (day0 is the start day of differentiation). The expression of cellular miR-199-3p was examined by qRT-PCR and analyzed by the delta-delta Ct method using the data of U6 snRNA as an internal reference. The level of exosomal miR-199-3p was examined as in Fig.1a. The data were normalized with the data of day0 as 1. Data are shown as mean±SEM (n = 3 independent determinations). (b) Effects of miR-199-3p inhibitor on the differentiation of C2C12 cells. MiRCURY LNA miRNA Inhibitors for miR-199-3p and a negative control were introduced to C2C12 cells by electroporation. The cells were cultured in differentiation medium as in a. 2 days later, total RNA was extracted. The expression levels of *Ckm*, *Myh1*, *Myog* as myogenic differentiation makers were examined by qRT-PCR and analyzed by the delta-delta Ct method using the data of *Gapdh* as an internal reference. The normalized data were further normalized to the data of negative control as 1. Data are shown as mean ±SEM (n = 3 independent determinations; $*p<0.05$, $**p<0.01$).

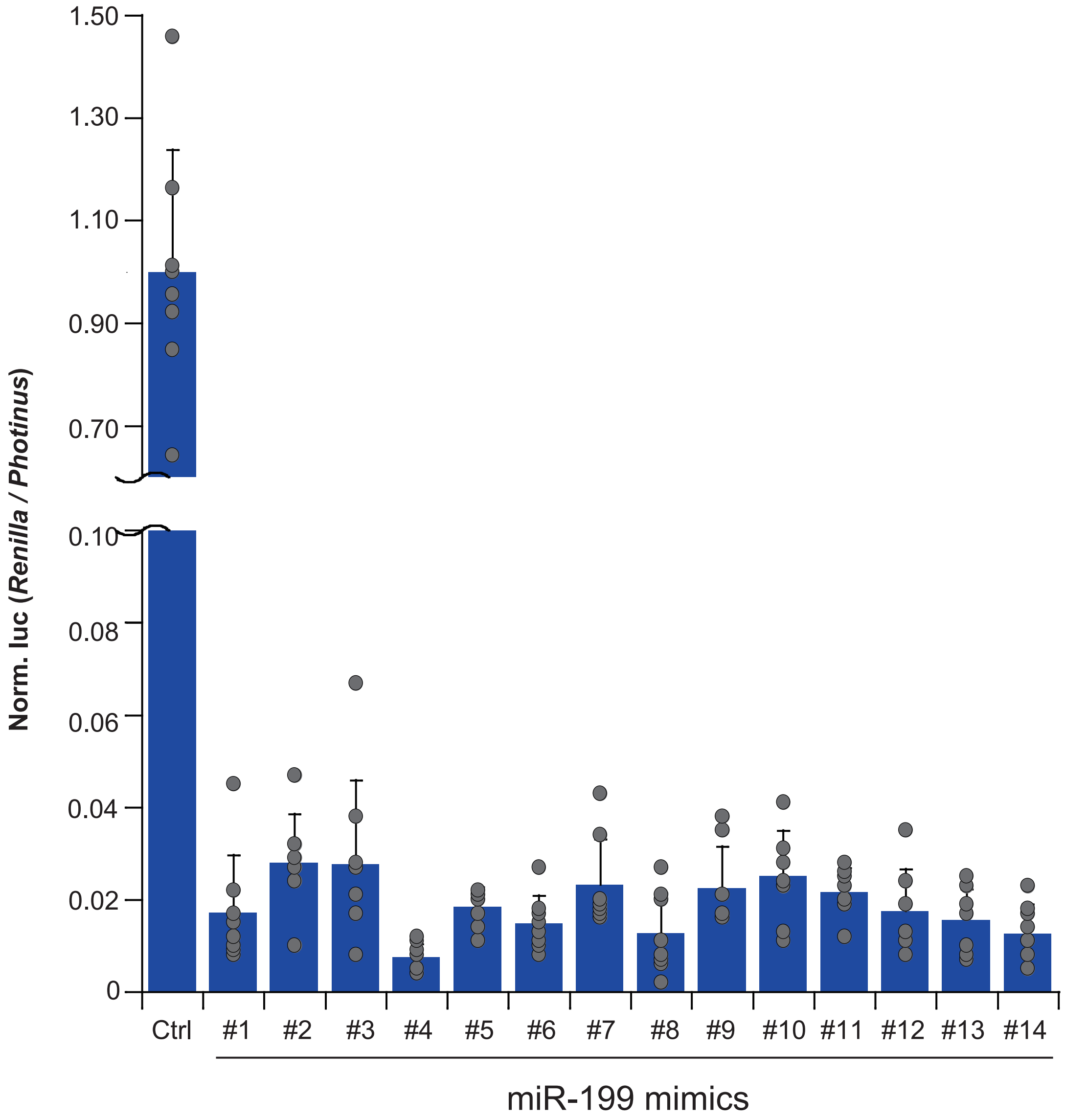

Supplementary Fig. 3. Screening of designed miR-199 mimics. 14 miR-199 mimics (#1~#14) were designed and chemically synthesized (Supplementary Table 1). Synthetic miR-199 mimics were transfected together with a reporter plasmid encoding the *Renilla luciferase* gene carrying the miR-199-3p complementary sequence to mouse Neuro2a cells. 24h after transfection, dual-luciferase assay was performed. The activity of the *Renilla* luciferase (target) was normalized to that of the *Photinus* luciferase, and further normalized to the data obtained from the cells treated with non-silencing control RNA duplex (Ctrl) as 1. Data are shown as mean±SEM (n = 8 independent determinations). Based on the data, we have selected the #4 mimic as a competent miR-199 mimic and named it 'miR199#4'.

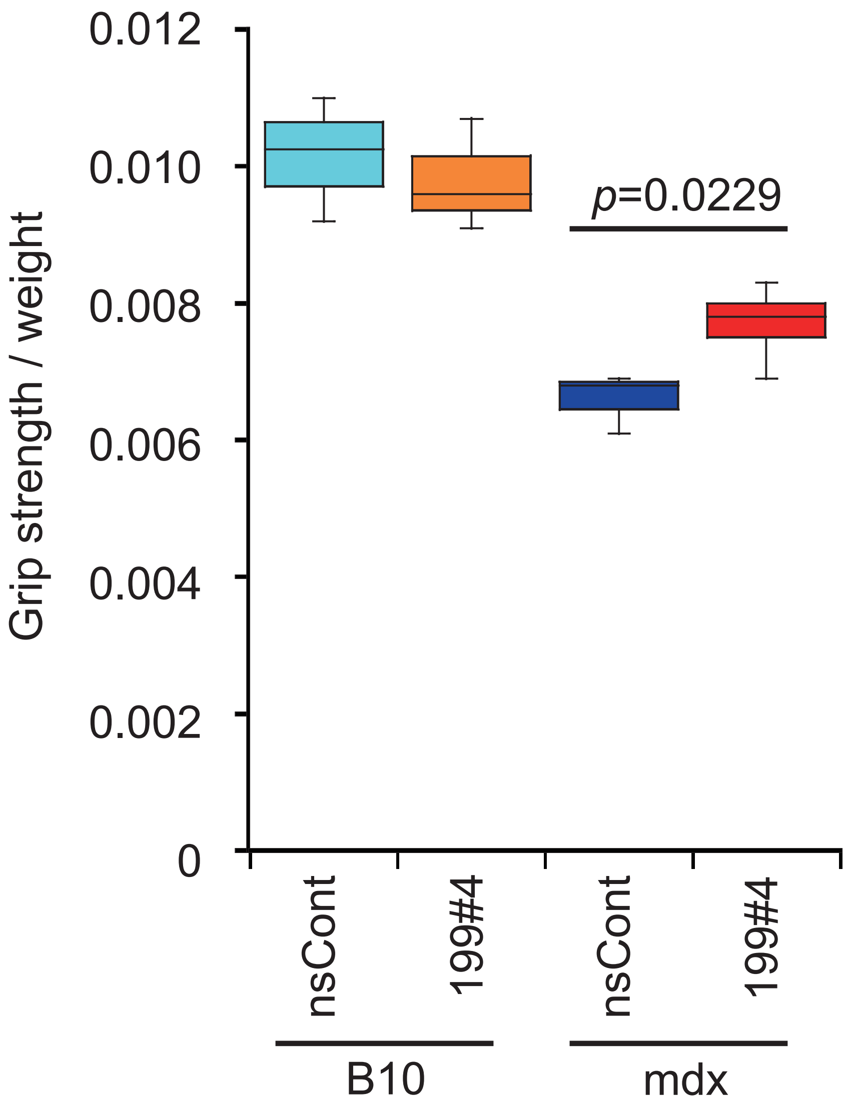

Supplementary Fig. 4. Treatment of mdx mice with miR199#4 using a new DDS reagent. Mdx and B10 mice (8-week-old) were injected twice intravenously with miR199#4 (199#4) or nsCont using DC-CHOL/DOPE cationic liposomes instead of atelocollagen, and examined by the grip strength test as in Fig. 8. The number of mice examined was as follows: 5 miR199#4-dosed mdx mice, 3 nsCont-dosed mdx mice, 3 miR199#4-dosed B10 mice, 4 nsCont-dosed B10 mice. Data are shown as mean±SEM.

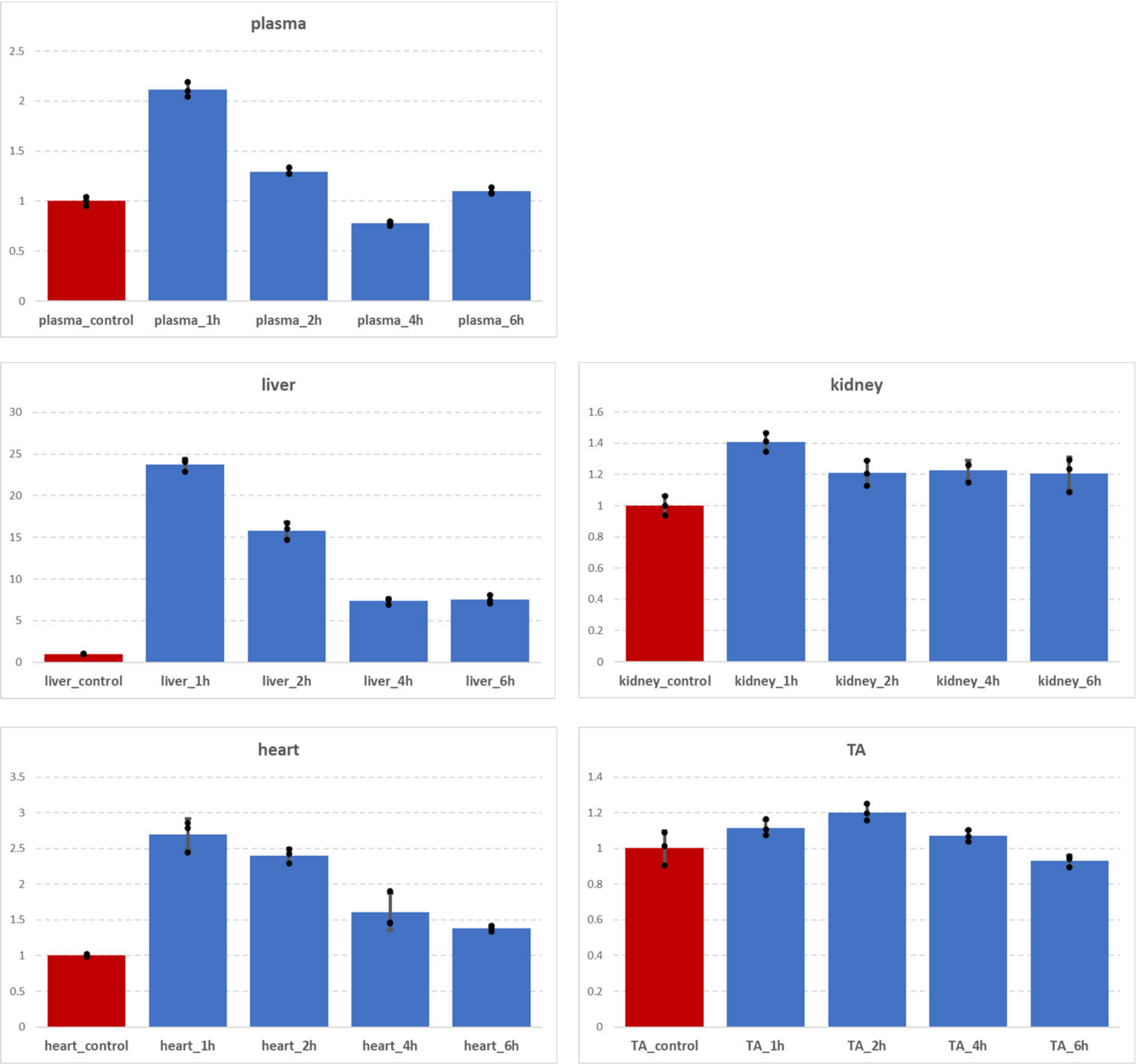

Supplementary Fig. 5. Drug distribution. MiR199#4 was intravenously administrated to C57BL/6J mice using DC-CHOL/DOPE cationic liposomes as in Supplementary Fig.4, and total RNA was extracted from the tissues at the indicated times after administration. The level of miR-199-3p was examined by qRT-PCR. The data were normalized to the data of miR-24 as an internal reference, and further normalized with the control data (injected with saline) as 1. Data are shown as mean ± SEM (n = 3 determinations).

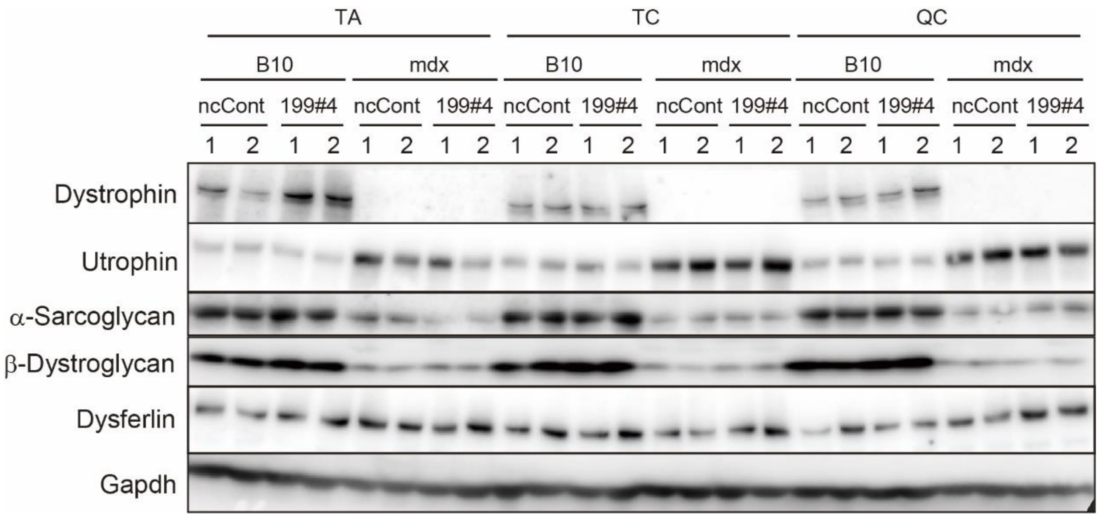

Supplementary Fig. 6. Western blotting. Mdx and B10 wild-type mice were intravenously injected with miR199#4 (199#4) and nsCont and followed by the grip strength test as in Fig. 8a. Mdx mice that markedly improved muscle strength by miR199#4 treatment were selected and examined together with the same litter mdx mice which were treated with nsCont. 2 representative mice (indicated by 1 and 2) in each group were examined. Examined proteins are indicated. Gapdh was examined as an internal control. TA: Tibialis anterior, TC: Triceps, QC: Quadriceps.

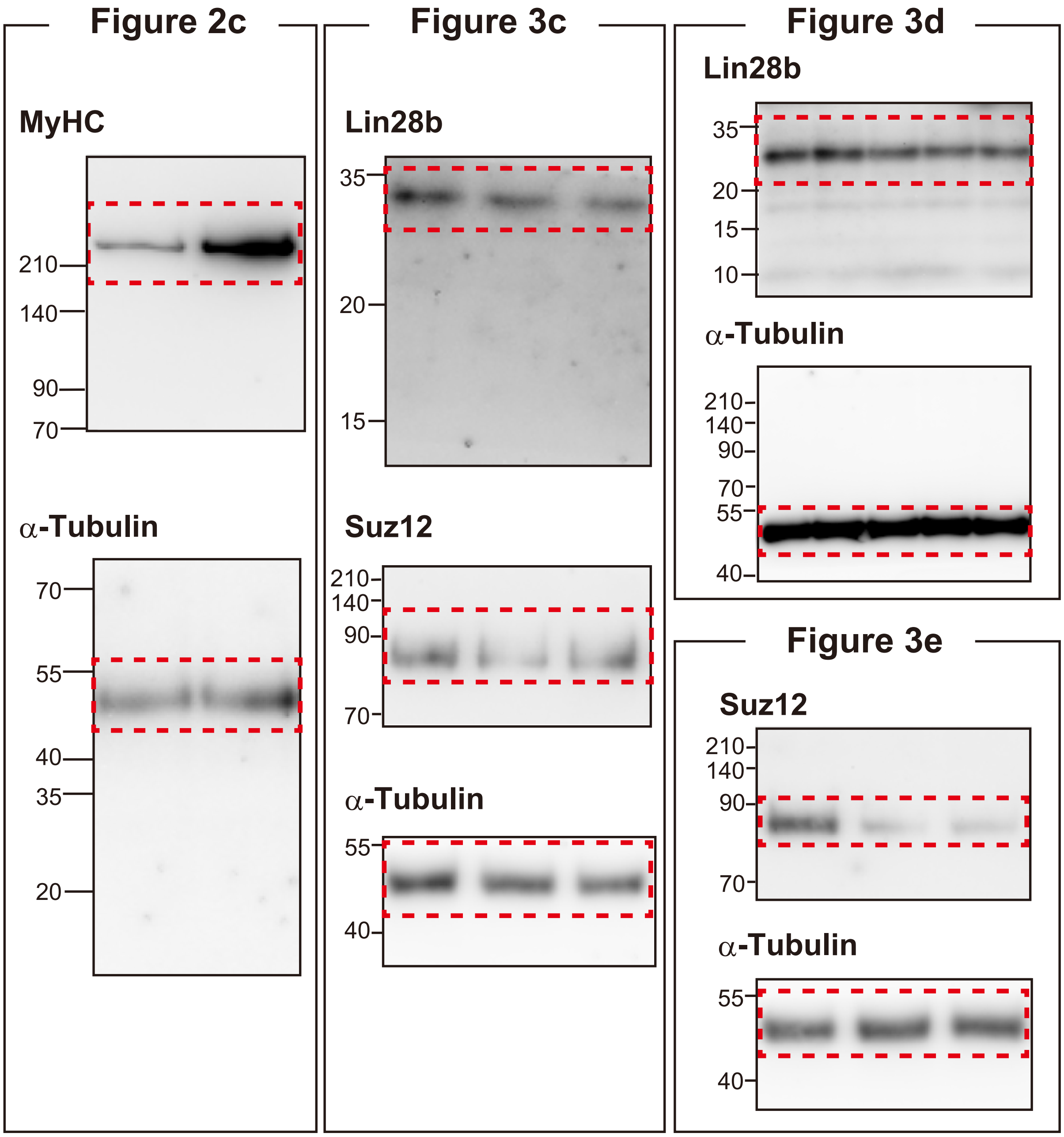

Supplementary Fig.7. Original blot images in Figs 2c and 3c-e. Blot images indicated by red boxes were used in Figures.

Supplementary Table 1. Sequences of siRNAs and miRNA mimics

Name	Strand*	Sequences (5'--3')
nsCont	SS	UUCUCCGAACGUGUCACGUUU
	AS	ACGUGACACGUUCGGAGAAUU
siSuz12-1	SS	ACUCGUCCAGGAAGAAGAGAAUUU
	AS	UAAAUUCUCUUCUUCCUGGACGAGU
siSuz12-2	SS	GAGAAUUUAAUGGAAUGAUUUU
	AS	AAUCAUUCCAUUAAAUUCUCUU
siLin28b-1	SS	GGAUUCAUCUCCAUGAUAAUU
	AS	UUAUCAUGGAGAUGAAUCCUU
siLin28b-2	SS	AGGAUUUAGAAGCUUGAAAUU
	AS	UUUCAAGCUUCUAAAUCCUUU
siLin28b-3	SS	GUGGAAUUUACAUUUAAAAUU
	AS	UUUUAAAUGUAAAUUCCACUU
siLin28b-4	SS	GAGCCAGUGGAAUUUACAUUU
	AS	AUGUAAAUUCCACUGGCUCUU
miR-199-SS**	SS	ACAGUAGUCUGCACAUUGGUUA
miR-199-AS#1	AS	ACCAAUGUGCAGACUACUGUUU
miR-199-AS#2	AS	ACCAAUGUGCAGACUAGUGUUU
miR-199-AS#3	AS	ACCAAUGUGCAGACUACCUGUUU
miR-199-AS#4	AS	ACCAAUGUGCAGACUACUCAUU
miR-199-AS#5	AS	CCCAGUGUUCAGACUACCUGUUU
miR-199-AS#6	AS	ACCAAUGUUCAGACUACCUGUUU
miR-199-AS#7	AS	CCCAGUGUUUAGACUAUCUGUUU
miR-199-AS#8	AS	ACCAAUGUUCAGACUACUCAUU
miR-199-AS#9	AS	ACCAAUGUGUAGAUUAUUCAUU
miR-199-AS#10	AS	ACCAAUGUGCAGAUUAUUCAUU
miR-199-AS#11	AS	ACCAAUGUGCAGACUAUUCAUU
miR-199-AS#12	AS	ACCAAUGUGUAGAUUACUCAUU
miR-199-AS#13	AS	ACCAAUGUGCAGAUUACUCAUU
miR-199-AS#14	AS	ACCAAUGUGUAGACUACUCAUU

* SS: sense strand, AS: antisense strand

** miR-199-SS is the miR-199-3p, and used as a sense-strand RNA and contained in all the miR-199 mimics.

Supplementary Table 2. Sequences of synthetic oligoDNAs

Name	Strand*	sequences (5'--3')
Lin28b (81)	SS	TCGAGCGGCCGGGATGTTAACTACTGCTTACTAGT
	AS	ACTAGTAAGCAGTAGTTAACATCCCGGCCGC
Lin28b (81)mut	SS	TCGAGCGGCCGGGATGTTATCATCAGCTTACTAGT
	AS	ACTAGTAAGCTGATGATAACATCCCGGCCGC
Lin28b (983)	SS	TCGAGAAGGTCTCTTACTTACTACTGATTACTAGT
	AS	ACTAGTAATCAGTAGTAAGTAAGAGACCTTC
Lin28b (983)mut	SS	TCGAGAAGGTCTCTTACTTACATGTCATTACTAGT
	AS	ACTAGTAATGACATGTAAGTAAGAGACCTTC
Suz12 (973)	SS	TCGAGCAGAAAGTGGTTTCACTACTGGTTACTAGT
	AS	ACTAGTAACCAGTAGTGAAACCACTTTCTGC
Suz12 (973)mut	SS	TCGAGCAGAAAGTGGTTTCACATGTCGTTACTAGT
	AS	ACTAGTAACGACATGTGAAACCACTTTCTGC
miR-199-3p	SS	TCGAGTAACCAATGTGCAGACTACTGTTTACTAGT
(perfect maching)	AS	ACTAGTAAACAGTAGTCTGCACATTGGTTAC

* SS: sense strand, AS: antisense strand

Oligonucleotides (SS & AS) indicated were annealed and inserted into the 3'UTR of *Renilla luciferase* in the psiCHECK-2 vector.
