# Supplementary material for: MiR-199-3p enhances muscle regeneration and ameliorates aged muscle and muscular dystrophy
Source: Commun Biol. 2021 Mar 29;4:427. doi: 10.1038/s42003-021-01952-2 (PMC8007565; doi:10.1038/s42003-021-01952-2)
Supplement: Supplementary file 2 — Description of Additional Supplementary Files [file 42003_2021_1952_MOESM2_ESM.pdf]

Description of Additional Supplementary Files

File name: Supplementary Data 1

Description: Source data for Main Figures.

File name: Supplementary Data 2

Description: Source data for Supplementary Figures.
