# Supplementary material for: MiR-199-3p enhances muscle regeneration and ameliorates aged muscle and muscular dystrophy
Source: Commun Biol. 2021 Mar 29;4:427. doi: 10.1038/s42003-021-01952-2 (PMC8007565; doi:10.1038/s42003-021-01952-2)
Supplement: Supplementary file 5 — Reporting Summary [file 42003_2021_1952_MOESM5_ESM.pdf]

nature research

Corresponding author(s): Hirohiko Hohjoh: COMMSBIO-20-2048A

Last updated by author(s): Feb 22, 2021

Reporting Summary

Nature Research wishes to improve the reproducibility of the work that we publish. This form provides structure for consistency and transparency in reporting. For further information on Nature Research policies, see our Editorial Policies and the Editorial Policy Checklist.

Statistics

For all statistical analyses, confirm that the following items are present in the figure legend, table legend, main text, or Methods section.

n/a	Confirmed	
☐	☒	The exact sample size (*n*) for each experimental group/condition, given as a discrete number and unit of measurement
☐	☒	A statement on whether measurements were taken from distinct samples or whether the same sample was measured repeatedly
☐	☒	The statistical test(s) used AND whether they are one- or two-sided<br>*Only common tests should be described solely by name; describe more complex techniques in the Methods section.*
☐	☒	A description of all covariates tested
☐	☒	A description of any assumptions or corrections, such as tests of normality and adjustment for multiple comparisons
☐	☒	A full description of the statistical parameters including central tendency (e.g. means) or other basic estimates (e.g. regression coefficient) AND variation (e.g. standard deviation) or associated estimates of uncertainty (e.g. confidence intervals)
☐	☒	For null hypothesis testing, the test statistic (e.g. F, t, r) with confidence intervals, effect sizes, degrees of freedom and P value noted<br>*Give P values as exact values whenever suitable.*
☒	☐	For Bayesian analysis, information on the choice of priors and Markov chain Monte Carlo settings
☒	☐	For hierarchical and complex designs, identification of the appropriate level for tests and full reporting of outcomes
☒	☐	Estimates of effect sizes (e.g. Cohen's *d*, Pearson's *r*), indicating how they were calculated

Our web collection on statistics for biologists contains articles on many of the points above.

Software and code

Policy information about availability of computer code

Data collection	N/A
Data analysis	The ImageJ software (a free-software) was used.

For manuscripts utilizing custom algorithms or software that are central to the research but not yet described in published literature, software must be made available to editors and reviewers. We strongly encourage code deposition in a community repository (e.g. GitHub). See the Nature Research guidelines for submitting code & software for further information.

Data

Policy information about availability of data

All manuscripts must include a data availability statement. This statement should provide the following information, where applicable:

- Accession codes, unique identifiers, or web links for publicly available datasets
- A list of figures that have associated raw data
- A description of any restrictions on data availability

The datasets generated during and/or analyzed during the current study are available in the Gene Expression Omnibus (GEO) repository (Accession number: GSE141258) in the NCBI. All data generated or analyzed during this study are including in this published article (and its supplementary information files), and the source data underlying the graphs are provided in Supplementary Data 1 and 2.

Field-specific reporting

Please select the one below that is the best fit for your research. If you are not sure, read the appropriate sections before making your selection.

[x] Life sciences   [ ] Behavioural & social sciences   [ ] Ecological, evolutionary & environmental sciences

For a reference copy of the document with all sections, see nature.com/documents/nr-reporting-summary-flat.pdf

Life sciences study design

All studies must disclose on these points even when the disclosure is negative.

Sample size	At least the number of samples that can be statistically analyzed (n ì 3) was examined.
Data exclusions	No data were excluded from the analyses.
Replication	To confirm the reproducibility, experiments with multiple samples and duplicated tests were carried out.
Randomization	For in vivo experiments with mice, mice were weighted before the test, and mice of similar weight were randomly divided into groups.
Blinding	In this study, the grip strength test was performed in a double-blind manner: RNA injection and the grip test were performed by different experimenters without sharing information.

Reporting for specific materials, systems and methods

We require information from authors about some types of materials, experimental systems and methods used in many studies. Here, indicate whether each material, system or method listed is relevant to your study. If you are not sure if a list item applies to your research, read the appropriate section before selecting a response.

Materials & experimental systems

n/a	Involved in the study
[ ]	[x] Antibodies
[ ]	[x] Eukaryotic cell lines
[x]	[ ] Palaeontology and archaeology
[ ]	[x] Animals and other organisms
[x]	[ ] Human research participants
[x]	[ ] Clinical data
[x]	[ ] Dual use research of concern

Methods

n/a	Involved in the study
[x]	[ ] ChIP-seq
[x]	[ ] Flow cytometry
[x]	[ ] MRI-based neuroimaging

Antibodies

Antibodies used	The primary antibodies used are indicated below with the product IDs and dilution ratios in parentheses:<br>Anti-Suz12 (#3737, 1/1000), anti-Lin28b (#5422, 1/1000) and anti-Gapdh (#2118, 1/5000) were purchased from Cell Signaling Technology. Anti-Myosin heavy chain (MF20) (MAB4470, 1/200) and anti-a-Tubulin (F2168, 1/5000) were purchased from R&D systems (Minneapolis, MN, USA) and Sigma-Aldrich, respectively. Anti-Dystrophin (ab15277, 1/1000), anti-a-Sarcoglycan (ab189254, 1/1000) and anti-Dysferlin (ab124684, 1/1000) were purchased from Abcam (Cambridge, UK). Anti-Utrophin (610896, 1/1000) and anti-β-Dystyroglycan (849401, 1/500) were purchased from BD Transduction Laboratories (Franklin Lakes, NJ, USA) and BioLegend (San Diego, CA, USA), respectively. Anti-Laminin-a2 (ALX-804-190, 1/200) was purchased from ENZO (Farmingdale, NY, USA).
Validation	All the primary antibodies used react with mouse antigens, and validation information for each antibody is available on the manufacturers' websites using the IDs indicated above.

Eukaryotic cell lines

Policy information about cell lines

Cell line source(s)	C2C12 cells were obtained from RIKEN BRC Cell Bank. Neuro2a cells are a cell line stored in our laboratory.
Authentication	None of the cell lines used were authenticated.
Mycoplasma contamination	The cell lines were not tested for mycoplasma contamination.
Commonly misidentified lines<br>(See ICLAC register)	n/a

Animals and other organisms

Policy information about studies involving animals; ARRIVE guidelines recommended for reporting animal research

Laboratory animals	C57BL/6J male mice, 6-week-old ~ >2-year-old; mdx male mice and B10 male mice, 8-week-old.
Wild animals	n/a
Field-collected samples	This study did not involve samples collected from the field.
Ethics oversight	All the animal experiments were performed in strict accordance with the recommendations in the Guide for the Care and Use of Laboratory Animals of the National Institutes of Neuroscience. The protocols were approved by the Committee on the Ethics of Animal Experiments of the National Institutes of Neuroscience (Permit Number: 20188005).

Note that full information on the approval of the study protocol must also be provided in the manuscript.
